# Addressing Pediatric HIV Pretreatment Drug Resistance and Virologic Failure in Sub-Saharan Africa: A Cost-Effectiveness Analysis of Diagnostic-Based Strategies in Children ≥3 Years Old

**DOI:** 10.3390/diagnostics11030567

**Published:** 2021-03-21

**Authors:** Mutita Siriruchatanon, Shan Liu, James G. Carlucci, Eva A. Enns, Horacio A. Duarte

**Affiliations:** 1 Department of Industrial & Systems Engineering, University of Washington, Seattle, WA 98185, USA; siriruc@uw.edu (M.S.); liushan@uw.edu (S.L.); 2 Department of Pediatrics, Ryan White Center for Pediatric Infectious Diseases and Global Health, Indiana University School of Medicine, Indianapolis, IN 46202, USA; jimcarlu@iupui.edu; 3 Division of Health Policy and Management, School of Public Health, University of Minnesota, Minneapolis, MN 55408, USA; eenns@umn.edu; 4 Department of Pediatrics, Division of Infectious Diseases, University of Washington, Seattle, WA 98105, USA; 5 Seattle Children’s Research Institute, Seattle, WA 98101, USA

**Keywords:** HIV, pretreatment drug resistance, regimen switching, drug resistance testing, NNRTI-based ART, dolutegravir-based ART, Africa, virologic failure

## Abstract

Improvement of antiretroviral therapy (ART) regimen switching practices and implementation of pretreatment drug resistance (PDR) testing are two potential approaches to improve health outcomes for children living with HIV. We developed a microsimulation model of disease progression and treatment focused on children with perinatally acquired HIV in sub-Saharan Africa who initiate ART at 3 years of age. We evaluated the cost-effectiveness of diagnostic-based strategies (*improved switching* and *PDR testing*), over a 10-year time horizon, in settings without and with pediatric dolutegravir (DTG) availability as first-line ART. The *improved switching* strategy increases the probability of switching to second-line ART when virologic failure is diagnosed through viral load testing. The *PDR testing* strategy involves a one-time PDR test prior to ART initiation to guide choice of initial regimen. When DTG is not available, PDR testing is dominated by the improved switching strategy, which has an incremental cost-effectiveness ratio (ICER) of USD 579/life-year gained (LY), relative to the status quo. If DTG is available, *improved switching* has a similar ICER (USD 591/LY) relative to the *DTG* *status quo*. Even when substantial financial investment is needed to achieve improved regimen switching practices, the *improved switching* strategy still has the potential to be cost-effective in a wide range of sub-Saharan African countries. Our analysis highlights the importance of strengthening existing laboratory monitoring systems to improve the health of children living with HIV.

## 1. Introduction

In sub-Saharan Africa, the prevalence of pretreatment drug resistance (PDR) to non-nucleoside reverse transcriptase inhibitor (NNRTI)-based antiretroviral therapy (ART) among children living with HIV is increasing, and PDR is associated with an increased risk of virologic failure [1,2]. In 2013, the World Health Organization (WHO) updated its guidelines to recommend protease inhibitor (PI)-based ART as the empiric first-line regimen for children initiating ART before 3 years of age [3,4,5]. However, these guidelines also continued to recommend NNRTI-based first-line ART for children initiating ART at 3 years of age or older [3]. Because PDR mutations persist as minority variants [6,7], this recommendation leaves children initiating ART in this older age group at increased risk of virologic failure.

Despite efforts to expand early infant diagnosis programs, nearly 40% of HIV-exposed infants are not retained in care long enough to definitively determine their HIV status [8]. As a result, some children living with perinatally-infected HIV, including those who are infected through breastmilk, go undiagnosed until they are 3 years or older. ART coverage in children living with HIV remains low (58% in eastern and southern Africa and 33% in western and central Africa) [9], and a substantial number of children likely initiate ART after 3 years of age [10,11,12,13]. This means that a substantial number of children are initiating NNRTI-based ART as their first-line regimen because they miss the age window for ART initiation with a PI-based regimen. Therefore, determining optimal diagnostic and treatment strategies to manage PDR and optimize treatment outcomes for this age group remains a priority.

Use of dolutegravir (DTG) is expected to address the challenges associated with PDR to NNRTI-based ART. Although DTG was approved for children in 2019 and is now recommended for use as first-line ART [14], its implementation in low- and middle-income countries (LMIC) has been limited to children weighing more than 20 kg [15], in effect making it available to only children aged 6 years and older [16]. This still leaves children 3 years and older but weighing less than 20 kg at increased risk of virologic failure due to the possibility of having PDR. While DTG formulations for young children weighing less than 20 kg are anticipated to become available in LMIC in the near future [17], the pace of scale-up is likely to vary depending on local health system considerations, with the potential for delayed implementation [18].

In children initiating ART who are 3 years and older, and for whom DTG is not currently an option, there are two diagnostic-based strategies that can potentially address the increased risk of virologic failure associated with PDR. First, introduction of PDR testing could be used to guide choice of first-line regimen, providing PI-based ART for children diagnosed with PDR. To date, the use of drug resistance testing for patient care in sub-Saharan Africa has been extremely limited due to multiple barriers, including the cost of testing and limited laboratory infrastructure [19]. Prior studies suggest PDR testing is unlikely to be cost-effective in adults [20,21,22,23], but similar analyses have not been conducted for children living with HIV. In addition to the prevalence of PDR being higher among children than adults [24], evidence suggests the increased risk of virologic failure associated with PDR may also be higher in children compared to in adults [2,25].

A second diagnostic-based strategy that can potentially improve health outcomes for children with or without PDR is to strengthen viral load testing systems, such that virologic failure is diagnosed in a more timely manner, and to facilitate a faster transition to second-line ART once virologic failure is detected [26]. In 2013, the WHO began recommending viral load testing as the preferred approach to monitor for treatment failure [3]. There is evidence that programs with viral load testing have higher rates of regimen switching compared to programs without viral load testing [27]. However, when compared to the relatively high rates of virologic failure observed among children in LMIC [28], the rate at which children with virologic failure switch to second-line ART appears to remain inappropriately low even when viral load testing is available [27,29,30]. Several factors are likely contributing to missed opportunities for regimen switching, including inadequate communication between clinics and laboratories regarding results; delayed turn-around times; insufficient human resources to meet high patient volume demands; concerns by clinicians about poor adherence to second-line ART; and, in some countries, additional approval requirements by health officials beyond the point of care [31,32]. Although DTG is expected to decrease rates of virologic failure once it is rolled out, similar challenges are likely to continue when virologic failure does occur if not explicitly addressed. Thus, it is important for ART programs and implementation scientists to develop a range of effective approaches to ensure that patients with virologic failure switch regimens in a timely fashion.

In this model-based analysis, we evaluated the cost-effectiveness of two diagnostic-based strategies, PDR testing and improved regimen switching practices when virologic failure is diagnosed on first-line ART, in the context of no dolutegravir availability for children initiating ART at 3 years of age. We then also evaluated the potential cost-effectiveness of improved regimen switching when an appropriate DTG-based ART formulation is available as the first-line regimen for children 3 years of age.

## 2. Materials and Methods

### 2.1. Overview

We developed a microsimulation model of disease progression and treatment focused on children with perinatally acquired HIV in sub-Saharan Africa who initiate ART at 3 years of age (see Supplemental Material Sections 1 and 4). Using this model, we simulated HIV disease progression, starting at birth, in a population with an 18% prevalence of PDR to NNRTI-based ART (Supplemental Material Section 2B) [1]. We assume that HIV diagnosis and ART initiation occurs at 3 years of age, and initial viral load testing is performed 6 months after ART initiation and then at 12-month intervals thereafter. For children who survive to the age 3 years, we first modeled comparative clinical and cost-effectiveness outcomes of the *status quo* and two diagnostic-based strategies in a scenario in which pediatric DTG is not available, over a 10-year time horizon. In the *status quo* strategy, we assumed that, under current clinical practice, a child diagnosed with virologic failure while on NNRTI-based first-line ART has a 40% probability of switching to PI-based second-line ART (see Supplemental Material Section 2A). In the *improved switching* strategy, we assumed a child diagnosed with virologic failure while on NNRTI-based first-line ART has an 80% probability of switching to PI-based second-line ART (double compared to *status quo*). Finally, in the *PDR testing* strategy, all children are tested for PDR prior to ART initiation, and children diagnosed with PDR initiate ART with a PI-based regimen.

Next, in a separate scenario, we evaluated the cost-effectiveness of diagnostic-based strategies when a pediatric DTG formulation is available and replaces NNRTIs in first-line ART. In this scenario, under the *DTG status quo* strategy, we assumed DTG-based ART is used as the first-line regimen, which is associated with a lower probability of virologic failure than NNRTI-based ART, and a child diagnosed with virologic failure while on first-line ART has a 40% probability of switching to PI-based second-line ART. In the *DTG improved switching* strategy, we assumed a child diagnosed with virologic failure while on DTG-based first-line ART has an 80% probability of switching to PI-based second-line ART (double compared to *DTG status quo*). Because rates of PDR to DTG are currently low, we omitted the PDR testing strategy and only evaluated the comparative clinical and cost-effectiveness of improvements of ART regimen switching practices when virologic failure is diagnosed (*DTG improved switching*) against the *DTG status quo*.

### 2.2. HIV Model

We developed a microsimulation model of disease progression and treatment for children living with HIV, starting at time of birth. Our pediatric HIV microsimulation model was based on an adaptation of a previously published model for adult HIV [23]. It was calibrated to survival outcomes among children (0–60 months old) living with HIV without access to treatment [33,34], as well as rates of opportunistic infection and mortality among children on ART [4,5,35]. Clinical, epidemiologic, and cost parameters were selected to be broadly representative of settings in sub-Saharan Africa. Key model parameter values are shown in Table 1, and a full, detailed description of our model is available in the Supplemental Material. Each child has several individual-level characteristics, including age, CD4%/CD4 cell count, ART regimen status, and PDR status, which are updated at monthly time cycles. Similar to a previously developed pediatric HIV microsimulation model [34,35], CD4% is used for children <5 years of age, and absolute CD4 cell count is used for children ≥5 years [36]. In the absence of effective ART, CD4%/CD4 cell count gradually decreases over time. A child’s risk of acute clinical events, including opportunistic infections, and HIV-related death are stratified by age and CD4%/CD4 cell count [37,38], and children can also die from non-HIV-related causes [39]. Once a child initiates effective ART, their CD4%/CD4 cell count gradually increase over time, and effective ART also decreases their risk of acute clinical events and HIV-related death.

### 2.3. ART Regimen Assumptions

We assumed that the probability of virologic failure during the first 12 months of ART is higher for children with PDR if they are initially treated with NNRTI-based ART (64.1% probability of virologic failure) compared those with PDR who receive PI-based ART (19.2% probability of virologic failure) and those who do not have PDR and are initially treated with NNRTI-based ART (19.2% probability of virologic failure). These model parameters assume the odds of virologic failure with initial ART are 7.5 times higher (odds ratio = 7.5) for those with PDR receiving an NNRTI-based regimen compared to those without PDR or those with PDR receiving PI-based ART as their initial regimen (see Supplemental Material Sections 2C and 2D). We assumed the probability of virologic failure on DTG-based first-line ART was 9.1% on the basis of extrapolation from adult data on the effectiveness of DTG-based ART relative to NNRTI-based ART (see Supplemental Material Section 2D) [40]. We assumed a child’s PDR status does not alter their probability of virologic failure with DTG-based ART. NNRTI-based ART and PI-based ART were estimated to cost USD 123/person/year and USD 290/person/year, respectively. Although they are not yet available, we assumed that pediatric formulations of DTG-based ART for children <20 kg would cost the same as NNRTI-based ART, given that these 2 regimens have similar costs in adults [43].

### 2.4. Health and Economic Outcomes

For each strategy modeled, we projected several health outcomes, including proportion of children with viral suppression, proportion of children surviving over time, person-months of ART use, proportion of children using PI-based ART, and total life years (LYs) accrued. To evaluate cost-effectiveness, we also considered the total direct health care costs incurred, reported in 2020 USD. Incremental cost-effectiveness ratios (ICERs) were calculated in terms of USD/LY gained. We adopted a health sector perspective, discounted future costs and benefits 3% annually, and adhered to the recent recommendations of the Second Panel on Cost-Effectiveness in Health and Medicine [49].

### 2.5. Sensitivity Analyses

To address uncertainty associated with epidemiologic, clinical, and cost parameters, we conducted one-way sensitivity analyses for multiple parameters, including PDR prevalence, probability of virologic failure with PDR on NNRTI-based ART, probability of virologic failure on PI-based second-line ART, and the costs of various components of HIV care (Table 1). Of note, while there are robust data to inform the probability of virologic failure with PI-based second-line ART for settings in which it is used after virologic failure on NNRTI-based first-line ART [41], data are extremely limited for settings in which PI-based second-line ART is used after virologic failure on DTG-based first-line ART. To account for the possibility that children who fail DTG-based ART may also have high rates of virologic failure on PI-based ART due to poor adherence with both regimens, we extended the range of virologic failure probabilities over 24 months on PI-based ART after DTG-based ART explored in sensitivity analysis to include less optimistic values (13.9–40.0% probability of virologic failure) compared to after NNRTI-based ART (13.9–19.4% probability for virologic failure; Table 1).

### 2.6. Scenario Analyses

In the base-case and sensitivity analyses described above, strategies with increased probability of switching to second-line ART when virologic failure is diagnosed (*improved switching* or *DTG improved switching*) do not incur any additional costs to achieve this improved switching, other than the additional cost of PI-based ART relative to first-line ART. However, in reality, any potential intervention designed to address this issue is likely to require financial investment. While some effective interventions have been identified to facilitate timely transition to second-line ART [50], more research is needed to develop a range of effective interventions, as barriers to timely regimen switching may vary depending on local contexts. In order to provide preliminary guidance to implementation scientists in developing these interventions, we conducted a targeted sensitivity analysis where we evaluated how the cost-effectiveness of improved switching strategies changed over a range of intervention costs (per child diagnosed with virologic failure) and levels of effectiveness (improvement in the probability of switching to second-line ART when virologic failure is diagnosed compared to the *status quo*).

## 3. Results

### 3.1. Health Outcomes

When pediatric DTG was not available, the proportion of children with suppressed viral load at 5 years after ART initiation was highest with the *improved switching* strategy (66.2%; Table 2), followed by the *PDR testing* strategy (65.0%), and finally *status quo* (63.7%). Similarly, the proportion of children alive 5 years after ART initiation was highest with the *improved switching* strategy (71.2%), followed by the *PDR testing* strategy (71.0%), and finally *status quo* (69.3%). When DTG was available, the proportion of children with suppressed viral load at 5 years after ART initiation was higher with the *DTG improved switching* strategy than the *DTG* strategy (68.3% vs. 67.4%), as was the proportion of children alive 5 years after ART initiation (72.1% vs. 71.4%). Overall, viral suppression and survival outcomes were better when DTG was available compared to when it was not (Table 2).

### 3.2. Costs

**Figure 1:**
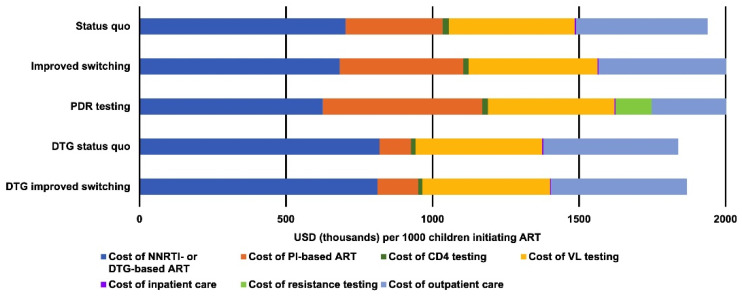
Total undiscounted costs for each strategy broken down by category. Costs are per 1000 children initiating ART over a 10-year time horizon.

Compared to the *status quo*, the *improved switching* and *PDR testing* strategies incurred 4% and 15% higher total costs, respectively (Figure 1). As these two diagnostic-based strategies had slightly higher survival rates than the *status quo*, the costs they accrued for viral load testing and outpatient visits, collectively, were slightly greater compared to the *status quo* (2.2% and 0.9% higher for *improved switching* and *PDR testing,* respectively). Person-months of ART use were also increased (4.70, 4.80, and 4.77 million person-months for *status quo*, *improved switching*, and *PDR testing*, respectively; Table 2). In addition, these two strategies also had a higher proportion of children using PI-based ART compared to the *status quo* (17%, 21%, and 26% for *status quo*, *improved switching*, and *PDR testing*, respectively), which costs more than NNRTI-based ART. For *improved switching*, increased ART costs made up 81% of the extra costs compared to the *status quo*. For *PDR testing*, increased ART costs and PDR testing made up 51% and 46% of the extra costs compared to the *status quo*, respectively.

Due to higher survival, using DTG as first-line ART for children 3 years of age (*DTG status quo*) resulted in 1.6% higher costs for viral load testing and outpatient visits, collectively, and more person-months of ART use (84.4 vs. 82.3 per person), compared to the *status quo*. However, higher rates of viral suppression with DTG were associated with less use of PI-based ART (4.5 person-months vs. 13.7 person-months per person) and a 5% decrease in total costs for *DTG status* quo compared to the *status quo*.

### 3.3. Cost-Effectiveness

**Figure 2:**
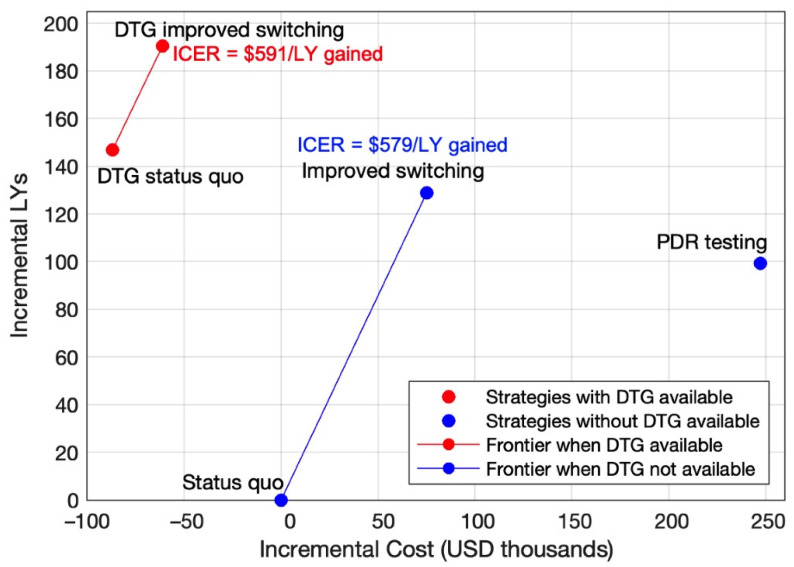
Incremental costs and health benefits of strategies compared to the *status quo*. Incremental costs and health benefits are per 1000 children initiating ART over a 10-year time horizon. No incremental cost-effectiveness ratio (ICER) was calculated for *pretreatment drug resistance* (*PDR*) *testing* because it was dominated by *improved switching* (*PDR testing* gained fewer life years (LYs) at a greater cost compared to *improved switching*).

Overall, strategies with DTG availability resulted in more LY’s gained compared to strategies without DTG availability (Figure 2; Table 3). When we only considered strategies in which DTG was not available, *improved switching* gained 131 additional discounted LYs (per 1000 children initiating ART) compared to the *status quo*, resulting in an ICER of USD 579/LY gained. *PDR testing* was dominated by *improved switching*, as *improved switching* achieved greater health gains (by 33 discounted LYs per 1000 children initiating ART) at a lower cost (by USD 171,167 per 1000 children initiating ART). When we only considered strategies with DTG availability, *DTG improved switching* gained 43 additional discounted LYs (per 1000 children initiating ART) compared to *DTG*, resulting in an ICER of USD 591/LY gained.

### 3.4. Sensitivity Analyses

**Figure 3:**
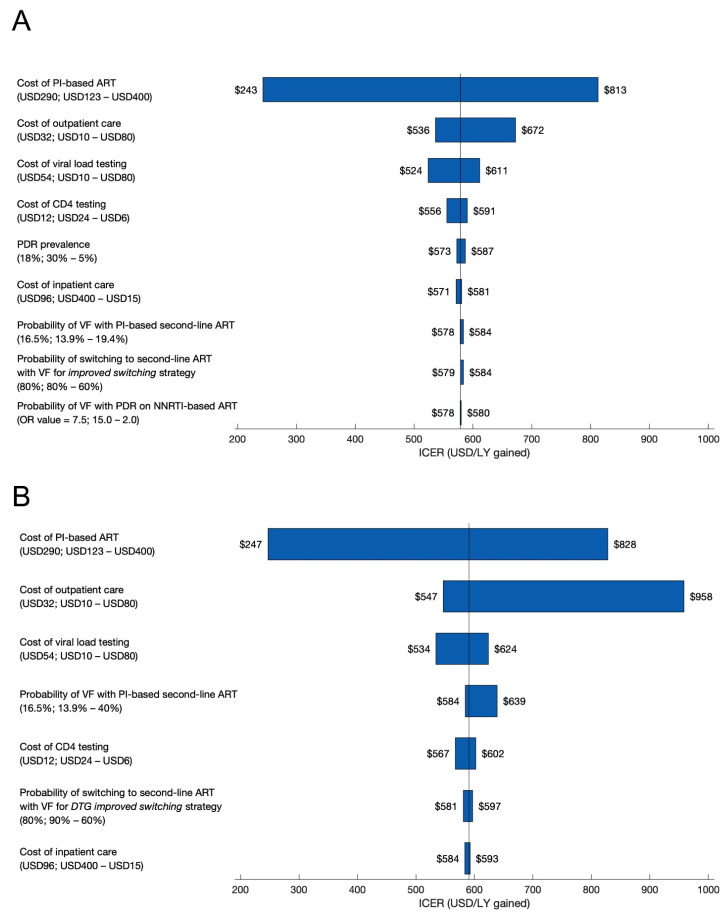
One-way sensitivity analyses of key model parameters. (**A**) *Improved switching* strategy. ICER values are for *improved switching* compared to *status quo*; the vertical bar represents the *improved switching* ICER from the base-case (USD 579/LY gained). (**B**) *DTG improved switching* strategy. ICER values are for *DTG improved switching* compared to *DTG status quo*; the vertical bar represents the *DTG improved switching* ICER from the base-case (USD 591/LY gained). Each horizontal bar presents the range of ICERs obtained when varying the corresponding single model parameter across the range of values we explored. The ranges of parameter values we explored are presented after each parameter label as (base-case value; parameter input value associated with lowest ICER—parameter input value associated with highest ICER). In the one-way sensitivity analysis of “probability of VF with PDR on NNRTI-based ART”, the odds ratio refers to the ratio of the odds of virologic failure for those with PDR on NNRTI-based ART compared to those with either no PDR on NNRTI-based ART or those with PDR on PI-based ART (“PDR to no PDR odds ratio”). Values for the probability of virologic failure corresponding to an odds ratio (OR) of 2.0 and an OR of 15.0 are explained in the footnotes of Table 1. VF = virologic failure; OR = odds ratio.

When we conducted one-way sensitivity analyses, the three parameters that had the greatest influence on the ICERs of the *improved switching* and *DTG + improved switching* strategies were the cost of PI-based ART, cost of outpatient care, and cost of viral load testing (Figure 3). Additionally, when we varied the effectiveness of the *improved switching* strategy, the *PDR testing* strategy could yield the most life-years gained (among strategies without DTG availability), but only when *improved switching* increased the probability of switching to PI-based second-line ART when virologic failure is diagnosed from 40% to only 60% or less (instead of 80%). However, this was achieved at a significantly higher cost per life-year gained (USD 10,466/LY gained for *PDR testing* vs. USD 584/LY gained for *improved switching* when probability of improved switching to PI-based second-line ART is 60% when virologic failure is diagnosed; Supplemental Material Section 5, Figure S3).

### 3.5. Scenario Analyses

**Figure 4:**
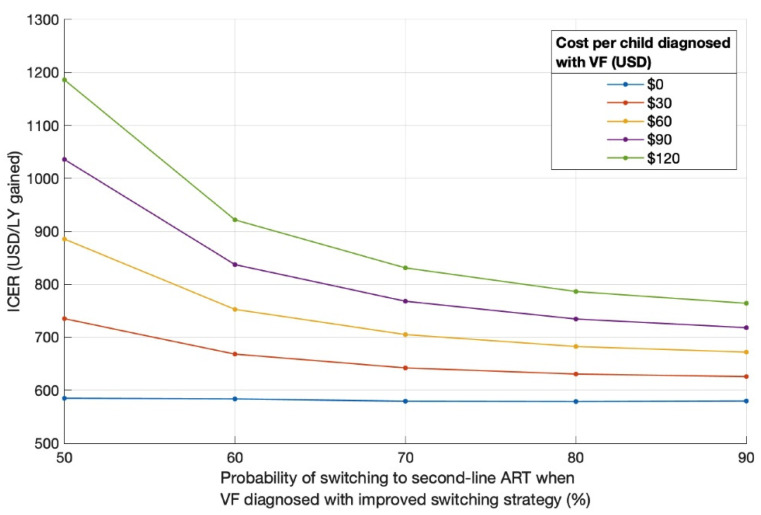
Cost-effectiveness of *improved switching* strategy relative to *status quo* over a range of strategy effectiveness and cost per child diagnosed with VF. VF = virologic failure.

For the *improved switching* strategy, if a program spent USD 120 per child diagnosed with virologic failure on a hypothetical intervention to increase the probability of switching to second-line ART when virologic failure was diagnosed, its ICER ranged from USD 764/LY gained (with a 90% probability of switching) to USD 1186/LY gained (with a 50% probability of switching) (Figure 4). When we conducted the same scenario analyses for the *DTG + improved switching* strategy, the range of ICERs was similar (range; Supplemental Material Section 5, Figure S4), and it increased slightly when we assumed the probability of virologic failure on PI-based ART after using DTG-based first-line ART was higher (40.0% instead of 16.4% over 24 months; Supplemental Material Section 5, Figure S5).

## 4. Discussion

As ART programs throughout sub-Saharan Africa await the availability of DTG for young children, improvement of ART regimen switching practices and implementation of PDR testing are two potential approaches to improve health outcomes in this population. We used a model-based analysis to evaluate the cost-effectiveness of these two diagnostic-based strategies in a setting without DTG availability for children initiating ART at 3 years of age. We also evaluated the potential cost-effectiveness of improved regimen switching when DTG-based ART was available as the first-line regimen.

In the context of no DTG availability, we found that improving regimen switching practices when virologic failure is diagnosed (increasing probability of switching to second-line ART from 40% to 80%) dominated PDR testing, with an ICER of USD 579/LY gained compared to the current standard of care (Figure 2; Table 3). These results are consistent with a prior model-based analysis in adults, which found that increasing the use of second-line ART was a more cost-effective strategy to address the challenges associated with PDR than testing for PDR prior to ART initiation [22]. Our analysis builds upon this prior work by evaluating the cost-effectiveness of a hypothetical intervention to improve regimen switching practices over a range of potential costs and levels of effectiveness. Even if improving regimen switching practices required an investment of USD 120 (i.e., the approximate cost of an entire year of NNRTI-based ART) per child diagnosed with virologic failure to improve the probability of switching to second-line ART to 70% (from 40%), such an intervention would potentially be cost-effective with an ICER of only USD 850/LY gained (Figure 4) compared to the standard of care. It is worth noting that that an ICER of USD 850/LY gained falls below the GDP per capita, a commonly used cost-effectiveness threshold [51], of a large portion of countries in sub-Saharan Africa [52]. As implementation scientists obtain data on the effectiveness and cost of newly designed interventions, our analysis can guide potential adjustments to increase their effectiveness, lower their cost, or both, such that their interventions are considered cost-effective in the local contexts where they are used. In contrast to PDR testing, which would require the introduction of an entirely new diagnostic system [19], efforts to improve regimen switching practices can begin now.

We found that the availability of DTG-based first-line ART provided greater health benefits than any of the non-DTG-containing strategies, thus supporting the importance of implementing DTG as soon as it is available for young children [17,18]. This finding is not surprising, as we extrapolated from adult data to assume DTG-based ART would achieve lower rates of virologic failure for all children, independent of their PDR status. Prior model-based analyses have shown similar findings in adults in LMIC [20,53]. We found that DTG-based ART could decrease overall costs due to lower rates of virologic failure on first-line ART and thus decreased need for PI-based ART. This finding was based on our assumption that DTG-based ART would cost the same as NNRTI-based ART, similar to adults. However, this assumption is uncertain as DTG formulations for young children and their cost estimates are not yet available. In addition, real-world estimates of rates of virologic failure on DTG-based first-line ART in LMIC are extremely limited in children. Importantly, the purpose of this analysis was not to evaluate whether DTG-based ART is cost-effective relative to NNRTI-based ART, but rather to examine potential strategies that can optimize ART prior to, and perhaps even after, roll-out of DTG for young children. As more DTG cost and real-world effectiveness data become available in the future, additional cost-effectiveness and budget impact analyses can be performed for specific settings.

Although rates of virologic suppression in children are expected to improve as DTG is used more widely, if not addressed, suboptimal regimen switching practices for patients with virologic failure are likely to persist even with DTG use. We found that potential interventions to improve regimen switching practices are likely to be similarly cost-effective when DTG-based ART is the first-line regimen compared to when NNRTI-based ART is the first-line regimen (Supplemental Material Section 5, Figure S4). One of the limitations of this finding is the lack of data to inform the probability of virologic failure on PI-based second-line ART after failing DTG-based first-line ART. However, even when we assumed a higher probability of virologic failure on PI-based second-line ART (40% vs. 16.4% over 24 months), the ICERs associated with this strategy were only slightly higher (Supplemental Material Section 5, Figure S5).

Once DTG becomes more widely available to children, more research will be needed to better understand potential risk factors for virologic failure with DTG-based ART in this population, such as nucleoside reverse transcriptase inhibitor (NRTI) backbone resistance. To date, evidence shows that PI-based ART achieves relatively high rates of viral suppression even with a non-active NRTI backbone, but such evidence is currently lacking for DTG-based ART [54]. If children have virologic failure with DTG-based ART that is functional monotherapy, due to NRTI resistance, switching to PI-based ART may, in fact, achieve relatively high rates of viral suppression. Although PDR testing may not be cost-effective, drug resistance testing could play an important role in guiding clinical decision making when virologic failure on DTG-based ART is diagnosed.

Our model also has some additional limitations. First, our broad focus on sub-Saharan Africa is intended to provide conceptual insights that are widely applicable in LMIC. However, each country will face its own particular considerations. For example, viral load testing may not be accessible for all children on ART, as we assumed. Also, baseline rates of switching to second-line ART when virologic failure is diagnosed are likely to vary by ART program. Second, our scenario analyses imply that the cost of potential interventions to improve regimen switching practices will be directly proportional to the number of children with virologic failure. As implementation scientists advance this work, they may find that some potential interventions experience economies of scale or diminishing returns as they scale-up. Additional analyses can be conducted and tailored to better meet the specifications of new interventions as they are developed. Third, our assumptions about the probability of virologic failure on DTG-based first-line ART are based on extrapolations from adult data. Last, our analysis focused only on children initiating ART at 3 years of age. However, the insights gained by our analysis regarding the potential cost-effectiveness of improving regimen switching practices are likely to have similar implications for children initiating ART at a younger age. Moreover, if implemented among adult men and women, improving switching practices could potentially decrease not only PDR among children but also the incidence of HIV among infants altogether.

In conclusion, while ART programs await the availability of DTG for young children, increased investment to improve regimen switching practices has the potential to be a cost-effective approach for improving health outcomes for children living with HIV. Our analysis provides economic data to further support ongoing calls to strengthen existing viral load testing systems to improve health outcomes [26]. Thus, our findings support investing in efforts to improve adherence to currently existing laboratory monitoring guidelines, rather than expanding or altering them. Interventions developed to improve regimen switching in the era of NNRTI-based ART have the potential to also be cost-effective once DTG becomes available. Finally, more research is needed to evaluate the potential role of drug resistance testing for patients diagnosed with virologic failure on DTG-based ART.

## Figures and Tables

**Table 1:** Model parameters for base-case analysis.

Parameter	Base-Case Estimate	Range for Sensitivity Analyses	Source
**Probability of virologic failure ^a^**			
Initial ART (over 12 months)			
No PDR on NNRTI-based ART	19.2%	16.8–24.6%	Boerma et al. [28], Kityo et al. [2]
PDR on PI-based ART	19.2%		
PDR on NNRTI-based ART	64.1%	39.5–75.2%	
Dolutegravir-based ART	9.1%		Boerma et al. [28], Dugdale et al. [40]
Second-line ART (over 24 months)			
PI-based ART after NNRTI-based first-line ART	16.4%	13.9–19.4%	Boerma et al. [41]
PI-based ART after DTG-based first-line ART	16.4%	13.9–40.0%	Assumption
**Cascade of care**			
*Status quo* probability of switching to second-line ART when virologic failure is diagnosed **^b^**	40%		Assumption
Probability of switching to second-line ART when virologic failure is diagnosed with improved regimen switching practices	80%	60.0–90.0%	
Probability of lost to follow-up (over 5 years) **^c^**	15%		Carlucci et al. [42]
**Unit Costs (USD) ^d^**			
ART annual cost			
NNRTI-based ART	$123		Global Fund [43]
Dolutegravir-based ART	$123		Assumption
PI-based ART	$290	$123–$400	Global Fund [43]
Inpatient day	$96	$15–$400 **^e^**	IHME [44,45,46,47], WHO-CHOICE [48]
Outpatient visit	$32	$10–$80	IHME [44,45,46,47], WHO-CHOICE [48]
CD4 testing	$12	$6–$24	Duarte et al. [23]
Viral load testing	$54	$10–$80	Duarte et al. [23]
Resistance testing	$125	$30–$250	Duarte et al. [23]

**^a^**Supplemental Material Sections 2C, 2D, and 2E discuss how sources were used to inform assumptions regarding probability of virologic failure on various antiretroviral therapy (ART) regimens. In the base-case scenario, the ratio of the odds of virologic failure for those with pretreatment drug resistance (PDR) on non-nucleoside reverse transcriptase inhibitor (NNRTI)-based ART compared to those with either no PDR on NNRTI-based ART or those with PDR on protease inhibitor (PI)-based ART is 7.5. We explored a one-way sensitivity analysis in which we varied the odds ratio from 2.0 to 15.0. An odds ratio of 2.0 corresponds to a probability of virologic failure for those with PDR on NNRTI-based ART of 39.5% and a probability of virologic failure for those with no PDR on NNRTI-based ART of 24.6%. An odds ratio of 15.0 corresponds to a probability of virologic failure for those with PDR on NNRTI-based ART of 75.2% and a probability of virologic failure for those with no PDR on NNRTI-based ART of 16.8%. **^b^** Supplemental Material Section 2A discusses data used to inform our assumption regarding the *status quo* probability of switching to second-line ART when virologic failure is diagnosed. **^c^** Supplemental Material Section 2F discusses how sources were used to inform assumptions regarding lost to follow-up. **^d^** Supplemental Material Section 3 discusses how sources were used to inform assumptions regarding unit costs. **^e^** This range is meant to capture uncertainty in both unit cost per inpatient day and the number of inpatient days per clinical event (see Supplemental Material Section 3B for details).

**Table 2:** Health and ART outcomes after ART initiation.

	Status Quo	Improved Switching	PDR Testing	DTG	DTG Improved Switching
**Health outcomes**					
Proportion of children with suppressed viral load at 5 years after ART initiation **^a^**	63.7%	66.2%	65.0%	67.4%	68.3%
Proportion of children alive at 5 years after ART initiation **^b^**	69.3%	71.2%	71.0%	71.4%	72.1%
**ART outcomes**					
Proportion of children on PI-based ART **^c^**	17%	21%	26%	5%	7%
Person-months of ART use (per person) **^d^**	82.3	84.1	83.5	84.4	85.0
Person-months of PI-based ART use (per person) **^d^**	13.7	17.4	22.5	4.5	5.7

**^a^** The numerator is the number of children with suppressed viral load at 5 years after ART initiation. The denominator includes all children who initiated ART at age 3 years, which by 5 years after ART initiation includes children with viral suppression, children who have been lost to follow-up, and children who have died. **^b^** The numerator is the number of children alive at 5 years after ART initiation. The denominator includes all children who initiated ART at age 3 years. **^c^** Average over 10-year time horizon. **^d^** Total (per-person) over 10-year time horizon.

**Table 3:** Costs, LYs, and incremental cost-effectiveness of strategies.

	**Undiscounted Cost (USD)**	**Undiscounted LYs**	
*Status quo*	1,938,996	7203	
*Improved switching*	2,025,987	7358	
*PDR testing*	2,203,694	7318	
*DTG*	1,838,619	7378	
*DTG + improved switching*	1,868,298	7430	
	**Discounted Cost (USD)**	**Discounted LYs**	**ICER (USD/LY gained)**
*Status quo*	1,697,253	6301	N/A
*Improved switching*	1,772,844	6432	579 **^a^**
*PDR testing*	1,944,011	6399	N/A
*DTG*	1,610,327	6448	N/A
*DTG + improved switching*	1,636,073	6491	591 **^b^**

Costs and LYs are per 1000 children initiating ART at 3 years of age, over a 10-year time horizon. Costs are reported in 2020 USD. Discounted costs and LYs were discounted 3% annually. N/A = not applicable; LYs = life years; ICER = incremental cost-effectiveness ratio. **^a^** ICER was calculated by considering only strategies without DTG availability. No ICER was calculated for *PDR testing* because it was dominated by *improved switching* (*PDR testing* gained fewer LYs at a greater cost compared to *improved switching*). **^b^** ICER was calculated by considering only strategies with DTG availability.

## Data Availability

The data presented in this study are openly available in FigShare at https://doi.org/10.6084/m9.figshare.14210609.

## Supplementary Materials

The following are available online at https://www.mdpi.com/2075-4418/11/3/567/s1: A Supplemental Materials document that includes an in-depth description of our pediatric HIV microsimulation model, various assumptions we made in our analyses, and supplemental figures that are referenced in this manuscript.

## References

[1] Boerma R.S., Sigaloff K.C., Akanmu A.S., Inzaule S., Boele van Hensbroek M., Rinke de Wit T.F., Calis J.C. (2017). Alarming increase in pretreatment HIV drug resistance in children living in sub-Saharan Africa: A systematic review and meta-analysis. J. Antimicrob. Chemother..

[2] Kityo C., Boerma R.S., Sigaloff K.C.E., Kaudha E., Calis J.C.J., Musiime V., Balinda S., Nakanjako R., Boender T.S., Mugyenyi P.N. (2017). Pretreatment HIV drug resistance results in virological failure and accumulation of additional resistance mutations in Ugandan children. J. Antimicrob. Chemother..

[3] WHO (2013). Consolidated Guidelines on the Use of Antiretroviral Drugs for Treating and Preventing HIV Infection: Recommendations for a Public Health Approach.

[4] Violari A., Lindsey J.C., Hughes M.D., Mujuru H.A., Barlow-Mosha L., Kamthunzi P., Chi B.H., Cotton M.F., Moultrie H., Khadse S. (2012). Nevirapine versus ritonavir-boosted lopinavir for HIV-infected children. N. Engl. J. Med..

[5] Palumbo P., Lindsey J.C., Hughes M.D., Cotton M.F., Bobat R., Meyers T., Bwakura-Dangarembizi M., Chi B.H., Musoke P., Kamthunzi P. (2010). Antiretroviral treatment for children with peripartum nevirapine exposure. N. Engl. J. Med..

[6] Kanthula R., Rossouw T.M., Feucht U.D., van Dyk G., Beck I.A., Silverman R., Olson S., Salyer C., Cassol S., Frenkel L.M. (2017). Persistence of HIV drug resistance among South African children given nevirapine to prevent mother-to-child-transmission. AIDS.

[7] Jain V., Sucupira M.C., Bacchetti P., Hartogensis W., Diaz R.S., Kallas E.G., Janini L.M., Liegler T., Pilcher C.D., Grant R.M. (2011). Differential persistence of transmitted HIV-1 drug resistance mutation classes. J. Infect. Dis..

[8] Carlucci J.G., Liu Y., Friedman H., Pelayo B.E., Robelin K., Sheldon E.K., Clouse K., Vermund S.H. (2018). Attrition of HIV-exposed infants from early infant diagnosis services in low- and middle-income countries: A systematic review and meta-analysis. J. Int. Aids Soc..

[9] UNAIDS Global HIV & AIDS Statistics—2020 Fact Sheet. https://www.unaids.org/en/resources/fact-sheet.

[10] Adedimeji A., Edmonds A., Hoover D., Shi Q., Sinayobye J.D., Nduwimana M., Lelo P., Nash D., Anastos K., Yotebieng M. (2017). Characteristics of HIV-Infected Children at Enrollment into Care and at Antiretroviral Therapy Initiation in Central Africa. PLoS ONE.

[11] Davies M.A., Phiri S., Wood R., Wellington M., Cox V., Bolton-Moore C., Timmerman V., Moultrie H., Ndirangu J., Rabie H. (2013). Temporal trends in the characteristics of children at antiretroviral therapy initiation in southern Africa: The IeDEA-SA Collaboration. PLoS ONE.

[12] Fatti G., Bock P., Eley B., Mothibi E., Grimwood A. (2011). Temporal trends in baseline characteristics and treatment outcomes of children starting antiretroviral treatment: An analysis in four provinces in South Africa, 2004-2009. J. Acquir. Immune Defic. Syndr..

[13] Sutcliffe C.G., Bolton-Moore C., van Dijk J.H., Cotham M., Tambatamba B., Moss W.J. (2010). Secular trends in pediatric antiretroviral treatment programs in rural and urban Zambia: A retrospective cohort study. BMC Pediatr..

[14] WHO (2019). Update of Recommendations on First- and Second-Line Antiretroviral Regimens.

[15] Bollen P.D.J., Moore C.L., Mujuru H.A., Makumbi S., Kekitiinwa A.R., Kaudha E., Parker A., Musoro G., Nanduudu A., Lugemwa A. (2020). Simplified dolutegravir dosing for children with HIV weighing 20 kg or more: Pharmacokinetic and safety substudies of the multicentre, randomised ODYSSEY trial. Lancet HIV.

[16] Jesson J., Desmonde S., Yiannoutsos C.T., Patten G., Malateste K., Duda S.N., Kumarasamy N., Yotebieng M., Davies M.A., Musick B. (2020). Weight-for-age distributions among children with HIV on antiretroviral therapy in the International epidemiology Databases to Evaluate AIDS (IeDEA) multiregional consortium. BMC Res. Notes.

[17] WHO (2020). Considerations for Introducing New Antiretroviral Drug Formulations for Children: Policy Brief.

[18] (2020). The Lancet HIV. End resistance to dolutegravir roll-out. Lancet HIV.

[19] Inzaule S.C., Ondoa P., Peter T., Mugyenyi P.N., Stevens W.S., de Wit T.F.R., Hamers R.L. (2016). Affordable HIV drug-resistance testing for monitoring of antiretroviral therapy in sub-Saharan Africa. Lancet Infect. Dis..

[20] Phillips A.N., Cambiano V., Nakagawa F., Revill P., Jordan M.R., Hallett T.B., Doherty M., De Luca A., Lundgren J.D., Mhangara M. (2018). Cost-effectiveness of public-health policy options in the presence of pretreatment NNRTI drug resistance in sub-Saharan Africa: A modelling study. Lancet HIV.

[21] Phillips A.N., Cambiano V., Miners A., Revill P., Pillay D., Lundgren J.D., Bennett D., Raizes E., Nakagawa F., De Luca A. (2014). Effectiveness and cost-effectiveness of potential responses to future high levels of transmitted HIV drug resistance in antiretroviral drug-naive populations beginning treatment: Modelling study and economic analysis. Lancet HIV.

[22] Nichols B.E., Sigaloff K.C., Kityo C., Hamers R.L., Baltussen R., Bertagnolio S., Jordan M.R., Hallett T.B., Boucher C.A., de Wit T.F. (2014). Increasing the use of second-line therapy is a cost-effective approach to prevent the spread of drug-resistant HIV: A mathematical modelling study. J. Int. Aids Soc..

[23] Duarte H.A., Babigumira J.B., Enns E.A., Stauffer D.C., Shafer R.W., Beck I.A., Garrison L.P., Chung M.H., Frenkel L.M., Bendavid E. (2020). Cost-effectiveness analysis of pre-ART HIV drug resistance testing in Kenyan women. EClinicalMedicine.

[24] WHO (2019). HIV Drug Resistance Report 2019.

[25] Hamers R.L., Schuurman R., Sigaloff K.C., Wallis C.L., Kityo C., Siwale M., Mandaliya K., Ive P., Botes M.E., Wellington M. (2012). Effect of pretreatment HIV-1 drug resistance on immunological, virological, and drug-resistance outcomes of first-line antiretroviral treatment in sub-Saharan Africa: A multicentre cohort study. Lancet Infect. Dis..

[26] Ehrenkranz P.D., Baptiste S.L., Bygrave H., Ellman T., Doi N., Grimsrud A., Jahn A., Kalua T., Nyirenda R.K., Odo M.O. (2019). The missed potential of CD4 and viral load testing to improve clinical outcomes for people living with HIV in lower-resource settings. PLoS Med..

[27] The Collaborative Initiative for Paediatric HIV Education, Research (CIPHER) Global Cohort Collaboration (2019). Incidence of switching to second-line antiretroviral therapy and associated factors in children with HIV: An international cohort collaboration. Lancet HIV.

[28] Boerma R.S., Boender T.S., Bussink A.P., Calis J.C., Bertagnolio S., Rinke de Wit T.F., Boele van Hensbroek M., Sigaloff K.C. (2016). Suboptimal Viral Suppression Rates Among HIV-Infected Children in Low- and Middle-Income Countries: A Meta-analysis. Clin. Infect. Dis..

[29] Wools-Kaloustian K., Marete I., Ayaya S., Sohn A.H., Van Nguyen L., Li S., Leroy V., Musick B.S., Newman J.E., Edmonds A. (2018). Time to First-Line ART Failure and Time to Second-Line ART Switch in the IeDEA Pediatric Cohort. J. Acquir. Immune Defic. Syndr..

[30] Davies M.A., Moultrie H., Eley B., Rabie H., Van Cutsem G., Giddy J., Wood R., Technau K., Keiser O., Egger M. (2011). Virologic failure and second-line antiretroviral therapy in children in South Africa--the IeDEA Southern Africa collaboration. J. Acquir. Immune Defic. Syndr..

[31] Murphy R.A., Court R., Maartens G., Sunpath H. (2017). Second-Line Antiretroviral Therapy in Sub-Saharan Africa: It Is Time to Mind the Gaps. Aids Res. Hum. Retrovir..

[32] Lecher S., Ellenberger D., Kim A.A., Fonjungo P.N., Agolory S., Borget M.Y., Broyles L., Carmona S., Chipungu G., De Cock K.M. (2015). Scale-up of HIV Viral Load Monitoring—Seven Sub-Saharan African Countries. Morb. Mortal. Wkly. Rep..

[33] Marston M., Becquet R., Zaba B., Moulton L.H., Gray G., Coovadia H., Essex M., Ekouevi D.K., Jackson D., Coutsoudis A. (2011). Net survival of perinatally and postnatally HIV-infected children: A pooled analysis of individual data from sub-Saharan Africa. Int. J. Epidemiol..

[34] Ciaranello A.L., Morris B.L., Walensky R.P., Weinstein M.C., Ayaya S., Doherty K., Leroy V., Hou T., Desmonde S., Lu Z. (2013). Validation and calibration of a computer simulation model of pediatric HIV infection. PLoS ONE.

[35] Ciaranello A.L., Doherty K., Penazzato M., Lindsey J.C., Harrison L., Kelly K., Walensky R.P., Essajee S., Losina E., Muhe L. (2015). Cost-effectiveness of first-line antiretroviral therapy for HIV-infected African children less than 3 years of age. AIDS.

[36] Department of Health and Human Services (2013). Guidelines for the Prevention and Treatment of Opportunistic Infections in HIV-Exposed and HIV-Infected Children. Panel on Opportunistic Infections in HIV-Exposed and HIV-Infected Children. J. Pediatric Infect. Dis. Soc..

[37] Ciaranello A., Lu Z., Ayaya S., Losina E., Musick B., Vreeman R., Freedberg K.A., Abrams E.J., Dillabaugh L., Doherty K. (2014). Incidence of World Health Organization stage 3 and 4 events, tuberculosis and mortality in untreated, HIV-infected children enrolling in care before 1 year of age: An IeDEA (International Epidemiologic Databases To Evaluate AIDS) East Africa regional analysis. Pediatric Infect. Dis. J..

[38] Desmonde S., Neilan A.M., Musick B., Patten G., Chokephaibulkit K., Edmonds A., Duda S.N., Malateste K., Wools-Kaloustian K., Ciaranello A.L. (2020). Time-varying age- and CD4-stratified rates of mortality and WHO stage 3 and stage 4 events in children, adolescents and youth 0 to 24 years living with perinatally acquired HIV, before and after antiretroviral therapy initiation in the paediatric IeDEA Global Cohort Consortium. J. Int. Aids Soc..

[39] United Nations, Department of Economic and Social Affairs Population Dynamics Population Division (2019). World Population Prospects 2019, Online Edition. https://population.un.org/wpp/Download/Standard/Population/.

[40] Dugdale C.M., Ciaranello A.L., Bekker L.G., Stern M.E., Myer L., Wood R., Sax P.E., Abrams E.J., Freedberg K.A., Walensky R.P. (2019). Risks and Benefits of Dolutegravir- and Efavirenz-Based Strategies for South African Women With HIV of Child-Bearing Potential: A Modeling Study. Ann. Intern. Med..

[41] Boerma R.S., Bunupuradah T., Dow D., Fokam J., Kariminia A., Lehman D., Kityo C., Musiime V., Palumbo P., Schoffelen A. (2017). Multicentre analysis of second-line antiretroviral treatment in HIV-infected children: Adolescents at high risk of failure. J. Int. Aids Soc..

[42] Carlucci J.G., Liu Y., Clouse K., Vermund S.H. (2019). Attrition of HIV-positive children from HIV services in low and middle-income countries. AIDS.

[43] Global Fund Global Fund Pooled Procurement Price List July 2020. https://www.theglobalfund.org/media/5813/ppm_arvreferencepricing_table_en.pdf.

[44] Institute for Health Metrics and Evaluation (IHME) (2014). Health Service Provision in Kenya: Assessing Facility Capacity, Costs of Care, and Patient Perspectives.

[45] Institute for Health Metrics and Evaluation (IHME) (2015). Health Service Provision in Ghana: Assessing Facility Capacity, Costs of Care, and Patient Perspectives.

[46] Institute for Health Metrics and Evaluation (IHME) (2014). Health Service Provision in Uganda: Assessing Facility Capacity, Costs of Care, and Patient Perspectives.

[47] Institute for Health Metrics and Evaluation (IHME) (2014). Health Service Provision in Zambia: Assessing Facility Capacity, Costs of Care, and Patient Perspectives.

[48] WHO-CHOICE Estimates of Unit Costs for Patient Services for South Africa. https://www.who.int/choice/country/zaf/cost/en/.

[49] Sanders G.D., Neumann P.J., Basu A., Brock D.W., Feeny D., Krahn M., Kuntz K.M., Meltzer D.O., Owens D.K., Prosser L.A. (2016). Recommendations for Conduct, Methodological Practices, and Reporting of Cost-effectiveness Analyses: Second Panel on Cost-Effectiveness in Health and Medicine. JAMA.

[50] Drain P.K., Dorward J., Violette L.R., Quame-Amaglo J., Thomas K.K., Samsunder N., Ngobese H., Mlisana K., Moodley P., Donnell D. (2020). Point-of-care HIV viral load testing combined with task shifting to improve treatment outcomes (STREAM): Findings from an open-label, non-inferiority, randomised controlled trial. Lancet HIV.

[51] WHO Commission on Macroeconomics and Health & World Health Organization (2001). Macroeconomics and Health: Investing in Health for Economic Development—Executive Summary.

[52] World Bank World Bank Open Data. https://data.worldbank.org/.

[53] Zheng A., Kumarasamy N., Huang M., Paltiel A.D., Mayer K.H., Rewari B.B., Walensky R.P., Freedberg K.A. (2018). The cost-effectiveness and budgetary impact of a dolutegravir-based regimen as first-line treatment of HIV infection in India. J. Int. Aids Soc..

[54] Vitoria M., Hill A., Ford N., Doherty M., Clayden P., Venter F., Ripin D., Flexner C., Domanico P.L. (2018). The transition to dolutegravir and other new antiretrovirals in low-income and middle-income countries: What are the issues?. AIDS.

