# Supplementary material for: Addressing Pediatric HIV Pretreatment Drug Resistance and Virologic Failure in Sub-Saharan Africa: A Cost-Effectiveness Analysis of Diagnostic-Based Strategies in Children ≥3 Years Old
Source: Diagnostics (Basel). 2021 Mar 21;11(3):567. doi: 10.3390/diagnostics11030567 (PMC8004076; doi:10.3390/diagnostics11030567)
Supplement: Supplementary file 1 [file diagnostics-11-00567-s001.pdf]

Table of Contents

Section 1. HIV Model *1*

A. Overview 1

B. Disease progression in children 0-4 years of age 1

C. Disease progression in children 5-13 years of age 2

D. ART and Immunologic Recovery 5

E. CD4% to CD4 cell count at age 5 years 5

Section 2. Epidemiologic, ART, and cascade of care assumptions *6*

A. Probability of switching to PI-based second-line ART when virologic failure is diagnosed 6

B. PDR prevalence 7

C. Risk of virologic failure associated with PDR 7

D. Probability of virologic failure on initial ART 7

E. Probability of virologic failure on PI-based second-line ART 9

F. Lost to follow-up 9

Section 3. Cost assumptions *10*

A. ART 10

B. Inpatient days 11

C. Outpatient visits 12

D. CD4 testing 12

E. Viral load testing 12

F. Resistance testing 13

Section 4. Model calibration *13*

A. Overview 13

B. Data sources used to inform parameter values 15

C. Calibration targets 17

i. Survival from birth to 60 months without ART 17

ii. Mortality and clinical event rates on ART 18

iii. CD4%/CD4 cell count conversion 19

Section 5. Additional results not included in manuscript *19*

References *21*

Section 1. HIV Model

A. Overview

We developed a microsimulation model of disease progression and treatment for children living with HIV, starting at time of birth. Our pediatric HIV microsimulation model was based on an adaptation of a previously published model for adult HIV [1]. Clinical, epidemiologic, and cost parameters were selected to be broadly representative of settings in sub-Saharan Africa. Briefly, each child has several individual-level characteristics, including age, CD4%/CD4 cell count, ART regimen status, and PDR status. These characteristics are updated at monthly time cycles. CD4% is used for children < 5 years of age, and absolute CD4 cell count is used for children ≥ 5 years [2]. In the absence of effective ART, CD4%/CD4 cell count gradually decrease over time. A child's risk of acute clinical events, including opportunistic infections, and HIV-related death are stratified by age and CD4%/CD4 cell count [3,4], and children can also die from non-HIV related causes [5], Once a child initiates effective ART, their CD4%/CD4 cell count gradually increase over time, and effective ART also decreases their risk of acute clinical events and HIV-related death. Each time we simulated a scenario with a unique set of parameter values (such as the base-case scenario or various sensitivity analysis scenarios), the simulation began running with 500,000 infants at birth, and the simulation was run 100 times. The results presented for each scenario represent the average of the results from the 100 runs conducted for each scenario.

In Section 1, we summarize and describe the final parameter values we used in our model, as briefly describe the sources we used to inform them. In Section 4, we provide a more detailed explanation of our model development and calibration process.

B. Disease progression in children 0-4 years of age

Initial CD4% and rate of CD4% decline

Model parameters that are relevant to disease progression for children 0-4 years of age in the absence of ART are summarized in **Table S1**. When an infant is born, they are assigned an initial CD4%, which is drawn from a normal distribution as described in **Table S1**. In the absence of ART, an infant's CD4% declines by 6.2% each month during the first 3 months of life and then by 0.5% each month through 5 years of age. Also, if a child initiates ART but then has virologic failure on their ART regimen, we assume their CD4% will decline while they are on an ineffective regimen. In our analysis, children do not initiate ART until 3 years of age.

Risk of clinical event and mortality

Each month, a child has some probability of having an acute clinical event or dying due to HIV-related causes. The monthly risk of a clinical event is stratified by age (< 6 months vs. > 6 months). Risk of death is stratified by age and CD4%. Once a child has a clinical event, their monthly risk of death is higher during the 30 days following the start of the clinical event, as well as beyond 30 days after the clinical event, compared to what their risk of HIV-related death would be if they did not have a history of a clinical event.

Our model parameters related to the probability of an acute clinical event and death were informed by two analyses conducted by Ciaranello et al., which, in this section, we refer to as "Ciaranello 2013" and "Ciaranello 2014" [3,6]. The Ciaranello 2014 analysis calculated age- and CD4%-stratified incidence of WHO Stage 3, WHO Stage 4, and tuberculosis (TB) events, as well as mortality, among children with perinatally-acquired HIV who were untreated and enrolled in care prior to 1 year of age. Data for these children came from seven different programs in Kenya, Uganda, and Tanzania that are a part of the International Epidemiologic Databases to Evaluate AIDS (IeDEA) East Africa consortium [3]. The authors describe that once ART was initiated, subsequent data, including events, death, and time at risk, was censored.

Ciaranello 2013 used data from the Ciaranello 2014 analysis to develop a pediatric HIV microsimulation model, known as the CEPAC-Pediatrics model, and this particular version of the model focused on disease progression in the absence of ART [6]. In contrast to the CEPAC-Pediatrics model, our model simulates the occurrence of clinical events as a whole, rather than WHO 3, WHO 4, and TB separately. Thus, we used the monthly probabilities presented by

Ciaranello 2013 in their Table 1 (based on data from Ciaranello 2014), converted them to monthly rates, took the average of these three monthly rates, and then converted this monthly average rate into the monthly probability of any clinical event.

The risk of clinical event and mortality as summarized in **Table S1** applies when a child is not on ART, or when a child is on ineffective ART and has virologic failure. Effective ART lowers the risk of clinical event and mortality, as described in Section 1D. In addition to the possibility of dying from an HIV-related cause, each child can also die from non-HIV related causes. Non-HIV related background mortality rates were based on data for sub-Saharan Africa [5], after subtracting the HIV-related component [7].

Table S1. Parameters relevant to disease progression for children 0-4 years old.

Parameters	Parameter Value		
Initial mean CD4% at birth (+/-SD)	44.2% (+/-10.0%)		
Monthly rate of CD4 decline			
Age ≤ 3 months	6.2%		
Age 4 months - 5 years	0.5%		
Monthly probability of a clinical event			
Age < 6 months	3.3%		
Age 6-59 months	4.6%		
Monthly probability of death with no history of clinical event	CD4%		
	< 15%	15-24%	> 25%
Age 0-6 months	6.3%	7.1%	6.9%
Age 7-12 months	4.6%	5.2%	5.0%
Age 13-24 months	1.5%	1.7%	1.6%
Age 25-36 months	1.6%	1.8%	1.8%
Age 37-48 months	0.4%	0.5%	0.5%
Age 49-60 months	0.1%	0.1%	0.1%
Probability of death within 30 days of clinical events			
Age < 5 years	11.1%		
Monthly probability of death with history of clinical event (> 30 days post-event)	CD4%		
	< 15%	15-24%	> 25%
Age 0-6 months	22.3%	7.9%	3.9%
Age 7-12 months	11.7%	4.0%	1.9%
Age 13-24 months	6.2%	2.1%	1.0%
Age 25-36 months	10.4%	3.5%	1.7%
Age 37-48 months	1.2%	0.4%	0.2%
Age 49-60 months	4.4%	1.5%	0.7%

To calculate the "monthly probability of a clinical event", we: 1) converted the monthly probability of a WHO Stage 3, WHO stage 4, and tuberculosis event (as presented in Table 1 of Ciaranello et al. [6]) each into a monthly rate; 2) took the average of these three rates to calculate an average monthly rate of a clinical event; 3) converted this monthly rate into a monthly probability, as presented in this table. With the exception of "monthly probability of a clinical event", all other parameters values were varied in our model calibration, described in detail in Section 4, and were informed by Ciaranello et al. [3,6]. SD = standard deviation. Initial mean CD4% above uses a normal distribution.

C. Disease progression in children 5-13 years of age

Rate of CD4 cell count decline

We assume the rate of CD4 cell count decline in the absence of ART (or with ineffective ART) among children 5 years or older is similar to what is observed among adults living with HIV, as described previously in Duarte et al. [1].

Risk of clinical event and mortality

For children who are 5-13 years old, their monthly probability of having a clinical event or an HIV-related death is summarized in **Table S2**. These probabilities were informed by data from Desmonde et al. [4], and a detailed explanation of how this data was used is provided in the subsequent sub-sections below.

Table S2. Parameters relevant to disease progression for children 5-13 years old.

Parameters	Parameter Value		
Monthly probability of clinical event			
Age 5-9 years	2.3%		
Age 10-14 years	1.0%		
Monthly probability of death with no history of clinical event	CD4 cell count		
	< 200	200-499	≥ 500
Age 5-9 years	0.2%	0.3%	0.3%
Age 10-14 years	0.1%	0.1%	0.1%
Probability of death within 30 days of clinical events			
Age 5-9 years	3.7%		
Age 10-14 years	2.1%		
Monthly probability of death with history of clinical event (> 30 days post-event)	CD4 cell count		
	< 200	200-499	≥ 500
Age 5-9 years	1.8%	0.6%	0.3%
Age 10-14 years	1.0%	0.3%	0.2%

In addition to the possibility of dying from an HIV-related cause, each child can also die from non-HIV related causes. Non-HIV related background mortality rates were based on data for sub-Saharan Africa [5], after subtracting the HIV-related component [7].

Derivation of probability of HIV-related death for children 5-13 years old

Table S3 summarizes the mortality incidence rates (per 100 person-years) found by Desmonde et al. for age groups 2-4 years, 5-9 years, and 10-14 years [4]. In the "Relative Risk" column, we derived the risk of death for age groups 5-9 years and 10-14 years, relative to the 2-4 year age group.

Table S3. Mortality risk for 5-9 and 10-14 year olds relative to 2-4 year olds.

Age (years)	Mortality IR (per 100 PY)	Relative Risk
2-4	3.40	reference
5-9	1.10	0.32
10-14	0.60	0.18

Data obtained from Table A of supplement for Desmonde et al. [4].

Table S4 provides a step-by-step guide of how we used the relative risks shown in **Table S3** to derive parameters on the probability of HIV-related death for children 5-13 years old, which includes: 1) probability of death with no history of clinical event, 2) probability of death within 30 days of a clinical event, and 3) probability of death with history of clinical event (>30 days post-event).

1. First, we converted parameters for HIV-related death for children ages 25-60 months (2-4 years), as presented above in **Table S1**, from monthly probabilities to monthly rates (rates can be multiplied and/or divided, but probabilities cannot).
2. We then calculated average monthly rates for these parameters for children ages 25-60 months (2-4 years), as shown in **Table S4**.
3. Next, we multiplied the monthly rates derived in step #2 by the relative risks for age groups 5-9 years and 10-14 years, in order to derive monthly rates of HIV related death for age groups 5-9 years and 10-14 years, respectively (results in **Table S4**).
4. Finally, we converted the monthly rates derived in step #3 to monthly probabilities (results in **Table S4**). These monthly probabilities are the same as those presented in **Table S2**.

Table S4. Derivation of probability of HIV-related death for children 5-13 years old.

RR = relative risk.

Age Group	Monthly rate of death with no history of clinical event			Rate of death within 30 days of OI	Rate of death with history of clinical event (> 30 days post-event)		
	CD4%				CD4%		
	<15%	15-24%	≥25%		<15%	15-24%	≥25%
Age 25-60 months	0.0073	0.0082	0.0080	0.1176	0.0556	0.0182	0.0087

	Monthly rate of death with no history of clinical event after multiplication by RR			Rate of death within 30 days of OI after multiplication by RR	Rate of death with history of clinical event (> 30 days post-event) after multiplication by RR		
	CD4 cell count				CD4 cell count		
	< 200	200-499	≥ 500		< 200	200-499	≥ 500
Age 5-9 years	0.0023	0.0026	0.0026	0.0380	0.0180	0.0059	0.0028
Age 10-14 years	0.0013	0.0014	0.0014	0.0208	0.0098	0.0032	0.0015

	Monthly probability of death with no history of clinical event			Probability of death within 30 days of clinical events	Monthly probability of death with history of clinical event (> 30 days post-event)		
	CD4 cell count				CD4 cell count		
	< 200	200-499	≥ 500		< 200	200-499	≥ 500
Age 5-9 years	0.23%	0.26%	0.26%	3.73%	1.78%	0.59%	0.28%
Age 10-14 years	0.13%	0.14%	0.14%	2.05%	0.98%	0.32%	0.15%

Derivation of probability of clinical event for children 5-13 years old

Table S5 summarizes the incidence rates (per 100 person-years) of WHO stage 3 and 4 clinical events found by Desmonde et al. for age groups 2-4 years, 5-9 years, and 10-14 years [4]. In the "Average IR" column, we derive the average of WHO 3 IR and WHO 4 IR. In "Relative Risk" column, we derived the risk of a clinical event for age groups 5-9 years and 10-14 years, relative to the 2-4 year age group.

Table S5. Risk of clinical event for 5-9 and 10-14 year olds relative to 2-4 year olds. Data obtained from Table A of supplement for Desmonde et al. [4].

Age (years)	WHO 3 IR (per 100 PY)	WHO 4 IR (per 100 PY)	Average IR (per 100 PY)	Relative Risk
2-4	9.40	3.20	6.30	reference
5-9	4.60	2.50	3.55	0.56
10-14	1.80	1.30	1.55	0.25

Table S6 provides a step-by-step guide of how we used the relative risks shown in Table S5 to derive the probability of having a clinical event for children 5-13 years old.

1. First, we converted parameters for probability of clinical event for children 0 to 59 months (0-4 years), as presented above in **Table S1**, from monthly probabilities to monthly rates (rates can be multiplied and/or divided, but probabilities cannot).
2. We then calculated the average monthly rate of a clinical event for children 0 to 59 months, as shown in **Table S6**.

3. Next, we multiplied the monthly rates derived in step #2 by the relative risks for age groups 5-9 years and 10-14 years, in order to derive monthly rates of clinical events for age groups 5-9 years and 10-14 years, respectively (shown in **Table S6**).
4. Finally, we converted the monthly rates derived in step #3 to monthly probabilities (shown in **Table S6**). These monthly probabilities are the same as those presented in **Table S2**.

Table S6. Derivation of probability of a clinical event for children 5-13 years old. RR = relative risk.

Parameter	Monthly rate of clinical event
Age 0-6 months	0.034
Age 6 - 59 months	0.047
Average	0.041
	Monthly rate of clinical event after multiplication by RR
Age 5-9 years	0.023
Age 10-14 years	0.010
	Probability of clinical events
Age 5-9 years	2.26%
Age 10-14 years	0.99%

D. ART and Immunologic Recovery

Once a child initiates effective ART, their CD4%/CD4 cell count increases each month, and they have a reduction in risk of clinical events and death, as described in **Table S7**.

Table S7. Parameters relevant to immunologic recovery on ART.

Parameters	Parameter Value
Monthly CD4%/absolute increase on effective ART	
Age < 5	
NNRTI first 6 months	2.3%
NNRTI after 6 months	0.7%
PI first 6 months	2.0%
PI after 6 months	0.4%
DTG first 6 months	2.3%
DTG after 6 months	0.7%
Age > 5	
NNRTI/PI/DTG first 6 months	67.3 cells/μL
NNRTI/PI/DTG after 6 months	3.4 cells/μL
Number of effective months for CD4	48
Reduction in event risk for patients on ART	
RRR of clinical event	0.88
RRR of mortality risk for age	0.97

Parameters related to monthly CD4% increase on NNRTI-based and PI-based ART for children < 5 years, as well as relative risk of having a clinical event and mortality were informed by Ciaranello et al.[8] and varied in our model calibration, described in detail in Section 4. Monthly increase in CD4 cell count while on NNRTI- or PI-based ART for children > 5 years were obtained directly from Ciaranello et al. [8]. We assumed monthly CD4% increase on DTG-based ART was the same as on NNRTI-based ART for children under 5 years old (and the same monthly CD4 cell count increase as NNRTI- or PI-based ART for children > 5 years. RRR = relative risk reduction.

E. CD4% to CD4 cell count at age 5 years

CD4% is used for children < 5 years of age, and absolute CD4 cell count is used for children ≥ 5 years [2]. For each child, once they reach 5 years of age, the model converts their CD4% value to a CD4 cell count value using the following algorithm:

1. First, for each child, the model calculates their percentile relative to all of their peers who are alive at that time point, who are all also 5 years old.
2. Second, a child with a CD4% value at the ith percentile relative to their peers is assigned a CD4 cell count value that corresponds to the ith percentile for the distribution described in **Table S8**, with the exception that CD4 cell count values below 100 are not assigned. For example, a 5 year-old child with a CD4% level at the 50%^{ile} relative to their peers is assigned a CD4 cell count that corresponds to the 50%^{ile} for the distribution described in **Table S8**.

Table S8. Initial CD4 cell count distribution used in CD4% to CD4 cell count conversion at age 5 years.

**Initial mean CD4 cell count at age 5 (SD)**	**547.2 (311.6)**

Our derivation for this CD4 cell count distribution parameter, which was adjusted in our model calibration, is described in detail in Section 4B. SD = standard deviation.

Section 2. Epidemiologic, ART, and cascade of care assumptions

A. Probability of switching to PI-based second-line ART when virologic failure is diagnosed

We assumed initial viral load testing is performed 6 months after ART initiation and is subsequently performed at 12-month intervals. We recognize that baseline rates of switching to second-line ART when virologic failure is diagnosed are likely to vary by country and ART program. Our assumption that there is a 40% probability of switching to PI-based second-line ART when virologic failure is diagnosed was informed by the data described below.

Evidence suggests that suboptimal rates of regimen switching may be widespread even when viral load testing is implemented. An international cohort study conducted by the Collaborative Initiative for Paediatric HIV Education and Research (CIPHER) Global Cohort Collaboration estimated the incidence of switching to second-line ART among children with HIV [9]. The cumulative incidence of switching to second-line ART by 3 years after ART initiation was 5.4% in Southern Africa among programs with routine CD4 and viral load testing. In the rest of sub-Saharan Africa, this incidence was 6.1% among programs with routine CD4 and viral load testing and 2.1% among programs with routine CD4 and targeted viral load testing. Although programs with viral load testing were associated with a higher incidence of regimen switching compared to programs without viral load testing, this incidence appears to be inappropriately low when compared to the relatively high rates of virologic failure observed among children in low- and middle-income countries (LMIC) [10]. Similar trends have been observed among adults living with HIV [11,12].

The CIPHER study noted whether or not patients were in ART programs with viral load testing availability; however, it did not note whether patients were actually diagnosed with virologic failure with a viral load test [9]. Thus, we examined additional pediatric HIV studies. First, a study including data from 7 different ART programs for children in South Africa, all with routine viral load testing, found that among 252 children with treatment failure who had at least 1 year of follow-up, only 38% of them were switched to second-line ART [13]. Second, in a retrospective, multi-country cohort study in sub-Saharan Africa, 4,763 children were found to have first-line ART failure, 45.4% of whom had treatment failure identified by virologic criteria [14]. These patients with ART failure were followed for a median of 14.3 months, and only 20.8% of them were switched to second-line ART. The study also found that routine viral load monitoring was not associated with a higher probability of transitioning to second-line ART after treatment failure.

In our model, when viral load testing reveals an unsuppressed viral load in a patient, a random number is generated for this patient, and if the random number is less than the assigned probability of switching to second-line ART (40% under the status quo; 80% under improved switching strategies), then the patient will switch to second-line ART. Otherwise, the patient will remain on first-line ART. If a patient is detected with virologic failure but is not switched to second-line ART at that time, they will be eligible for switching regimens the next time viral load

testing is performed (using the same algorithm with same probability corresponding to strategy), which we assume occurs 12 months later, although there is little data to guide this assumption.

B. PDR prevalence

We assume our model-based population has an 18% prevalence of PDR to non-nucleoside reverse transcriptase inhibitor (NNRTI)-based ART, which includes mutations conferring resistance to NNRTI or nucleoside reverse transcriptase inhibitors (NRTI) agents. This assumption is informed by a meta-analysis of studies on PDR in children in sub-Saharan conducted by Boerma et al., as well as the 2019 WHO Drug Resistance Report [15,16]. The Boerma et al. study had a few key findings. First, they found that the prevalence of PDR (which includes resistance to NNRTI or NRTI agents) in children has been increasing at an alarming rate, as the prevalence of PDR increased from 0% in 2004 to 26.8% in 2013. Second, when they pooled data by prevention of mother-to-child transmission (PMTCT) exposure status, they found PDR prevalence to be 42.7% among PMTCT-exposed children and 12.7% among PMTCT-unexposed children. Third, when the data was analyzed by age, they found the prevalence of PDR among children < 3 years old and ≥ 3 years old was 40.9% and 17.6%, respectively. Our model does not make any assumptions regarding the prevention of mother-to-child transmission (PMTCT) exposure status of the children in our simulation. Thus, we utilized the age-based data they provided and assumed the prevalence of PDR among children in our model initiating ART at 3 years of age was 18%. However, we recognize the PDR prevalence found by Boerma et al. among children > 3 years old may underestimate the true prevalence. This estimate likely reflects the transition of drug resistance mutations from majority to minority state over time in the absence of selective pressure, resulting in the mutant population falling below the level of detection of consensus sequencing [17].

Estimates of PDR prevalence in children were even higher in the 2019 WHO Drug Resistance Report, which provides estimates of PDR prevalence among newly diagnosed infants across 9 different countries in sub-Saharan Africa [16]. Even for PDR to NNRTI agents alone, in any given country, prevalence ranged from >10% to >30% among infants with no (or unknown) PMTCT exposure, and ranged >30% to >70% among infants with known PMTCT exposure. For NRTI PDR, in any given country, prevalence ranged from <5% to > 20% [16].

Thus, in our one-way sensitivity analysis of PDR prevalence, we explored values ranging from 5-30%.

C. Risk of virologic failure associated with PDR

We assume the odds of virologic failure with NNRTI-based first-line ART are 7.5 times higher (odds ratio = 7.5) for those with PDR (to at least one agent in NNRTI-based ART) receiving an NNRTI-based regimen compared to those without PDR. This assumption is informed by a study conducted by Kityo et al. in a population of children in Uganda, which found this odds ratio to be 15.25 (95% CI 3.77–61.67; P<0.001) [18]. This odds ratio estimate is markedly higher than estimates found in adults. For example, a multi-center cohort study conducted in six sub-Saharan African countries (including Kenya) found that compared to participants without PDR, the odds ratio for virologic failure was increased (OR = 2.13) in participants with PDR to at least one prescribed drug, but not in individuals with PDR and fully active ART [19]. Second, in a randomized controlled trial in Kenya, among subjects with PDR to first-line ART, those who underwent drug resistance testing and started PI-based ART had a 14.3% probability of failure at 12 months compared to 50.0% probability of failure at 12 months in those who were not tested for drug resistance and started NNRTI-based ART [20]. Expressed a different way, compared with subjects with PDR on PI-based ART, the odds ratio for virologic failure was 5.99 in subjects with PDR to at least one prescribed drug. Due to the relatively small sample size of children in the Kityo et al. study compared to adult studies, we decided to use a more conservative assumption in the base-case analysis and assumed the odds ratio was 7.5, instead of 15.25. In a one-way sensitivity analysis, we explored odd ratio values ranging from 2 to 15.

D. Probability of virologic failure on initial ART

Probability of virologic failure on NNRTI-based first-line ART

Our assumptions regarding the probability of virologic failure on NNRTI-based first-line ART were informed by a meta-analysis of viral suppression rates among children in low- and middle-income countries [10]. This study found that, among studies conducted in 2010 or later, 72.7% of children on ART after 12 months of ART achieve viral suppression (23.3% have virologic failure), in an on-treatment analysis [10]. We used data from their on-treatment analysis because our model accounts for being lost to follow-up and death. This data does not provide estimates of virologic failure rates based on PDR status, which is ultimately what we need to parameterize our model. Therefore, we adjusted this data based on two assumptions we made: 1) prevalence of PDR was 18%; and 2) the odds of virologic failure with NNRTI-based first-line ART are 7.5 times higher (odds ratio = 7.5) for those with PDR receiving an NNRTI-based regimen compared to those without PDR.

We used an algebraic approach to estimate the following parameters, where:

- X = probability of virologic failure on NNRTI-based ART without PDR (over initial 12 months on ART)
- Y = probability of virologic failure on NNRTI-based ART with PDR (over initial 12 months on ART)

Our algebraic approach included the following assumptions:

- Proportion of HIV-infected patients on ART with virologic failure = 23.3%
- PDR prevalence = 18%
- Odds ratio of the probability of virologic failure for patients with PDR to NNRTI-based first-line ART compared to patients without PDR to initial ART is 7.5

We solved for X and Y using the following 2 equations:

- 0.82X + 0.18Y = 0.233
- (Y/(1-Y)) / (X/(1-X)) = 7.5

We found X = 19.2% and Y = 64.1%.

Of note, to operationalize a 12-month probability of failure in the model, it is converted into a monthly probability through the following steps, where p = probability, t = time, and r = rate:

1) Convert the 12-month probability to a 1-month rate: $\mathbf{p = 1 - \exp^{(-rt)}}$**, where** $\mathbf{t = 12}$
2) Convert the 1-month rate to a 1-month probability: $\mathbf{r = (-\ln(1 - p))/ t}$**, where** $\mathbf{t = 1}$

Probability of virologic failure with PI-based first-line ART for children with PDR

In the *PDR testing* strategy, children diagnosed with PDR are started on a protease-inhibitor (PI)-based regimen. We assume their probability of virologic failure on this regimen is equal to the probability of virologic failure for children on NNRTI-based ART without PDR. This assumption is an extrapolation from results from a randomized trial in Kenyan adults in which patients with PDR who received a PI-based first-line ART regimen had similar rates of virologic failure compared to patients without PDR who received a NNRTI-based first-line ART [20]. The same assumption was made in a prior model-based analysis for adults living with HIV [1].

Probability of virologic failure with dolutegravir-based first-line ART

Estimates of rates of virologic suppression with dolutegravir-based ART among children in real-world settings are extremely limited. We used a recent literature review of adult clinical trial data to inform our assumptions about the relative risk of virologic failure with dolutegravir-based ART compared to efavirenz-based ART [21]. To parameterize their model-based analysis, Dugdale et al performed a literature review of randomized clinical trials reporting virologic suppression with dolutegravir or efavirenz in combination with two nucleoside reverse transcriptase inhibitors and then pooled these estimates (and weighted by study size). They estimated that the probabilities of viral suppression after 48-weeks on efavirenz-based (only subjects without PDR were eligible) and dolutegravir-based ART were 91% and 96%, respectively. Based on these estimates, for patients without NNRTI-associated PDR, the odds of virologic failure with efavirenz-based ART are 2.37 times higher (odds ratio = 2.37) than the odds

of virologic failure with dolutegravir-based ART. Although this was based on adult data, we assumed that a similar relationship would apply to pediatric populations. Based on this odds ratio of 2.37 and our prior assumption that the probability of virologic failure with NNRTI-based ART without PDR (during the first 12 months of ART) is 19.2%, we estimate that the probability of virologic failure with dolutegravir-based ART among children (during the first 12 months of ART) is 9.1%. We assume NNRTI-associated PDR does not influence the probability of virologic failure on dolutegravir-based ART.

Long-term probability of failure on first-line ART

In a meta-analysis, rates of viral suppression observed in children are similar 24 months after ART initiation compared to 12 months after ART initiation, and data on health outcomes beyond 24 months after ART initiation in children are scarce [10]. Thus, we made the decision to assume that if a child has not experienced virologic failure during the first 12 months of first-line ART that they will continue to maintain viral suppression on this regimen in the future.

E. Probability of virologic failure on PI-based second-line ART

Probability of virologic failure on PI-based second-line ART after being on NNRTI-based ART

This parameter was informed by an analysis that included 928 children from 12 cohorts from 14 different countries across sub-Saharan Africa and Asia [22].

Probability of virologic failure on PI-based second-line ART after being on DTG-based ART

Currently, WHO guidelines recommend switching to PI-based second-line ART when children have virologic failure on DTG-based ART [23]. Data to inform the probability of virologic failure on PI-based second-line ART after using DTG-based first-line ART are extremely limited for both adults and children in LMIC, and is limited even in high-income settings. This is not surprising given how recently programs in LMIC began implementing DTG-based ART. Thus, for the base-case analysis, we assumed the probability of virologic failure on PI-based second-line ART after being on DTG-based ART was the same as it is after being on NNRTI-based ART. To account for the possibility that children who fail DTG-based ART may also have high rates of virologic failure on PI-based ART due to poor adherence with both regimens, we extended the range of virologic failure probabilities over 24 months on PI-based ART after DTG-based ART explored in sensitivity analysis to include less optimistic values (13.9 – 40.0% probability of virologic failure) compared to after NNRTI-based ART (13.9 – 19.4% probability for virologic failure; Table 1).

F. Lost to follow-up

We assume 15% of children who initiated ART were lost to follow-up (LTFU) over the course of 5 years. This assumption was informed by a systematic review and meta-analysis conducted by Carlucci et al. in children in low- and middle-income countries, primarily in Africa [24]. Data summarized in their Figure 2 found that after ≤6 months, >6 to ≤12 months, >12 to ≤24 months, >24 to ≤36 months, >36 to ≤48 months, and >48 to ≤60 months of care, the proportion of children who were LTFU were 11.1%, 11.3%, 16.6%, 13.5%, 10.7%, and 9.5%, respectively. Although we recognize that in real-world settings LTFU most likely continues beyond 5 years of care, we assumed no further LTFU occurred if a patient remained in care after 5 years, due to lack of data beyond this time period. We chose 15% LTFU over 5 years to reflect the highest values observed over time (namely in the >12 to ≤24-month time period) even though only 9.5% LTFU was observed among studies with data during the >48 to ≤60 time period. We assume that children who remain in care continue to take ART, while children who are LTFU stop taking ART.

Of note, to operationalize a 15% probability of LTFU over 5 years in the model, it is converted into a monthly probability through the following steps, where p = probability, t = time, and r = rate:

1) Convert the 12-month probability to a 1-month rate: $\boldsymbol{p = 1 - \exp^{(-rt)}}$**, where** $\boldsymbol{t = 12}$
2) Convert the 1-month rate to a 1-month probability: $\boldsymbol{r = (-\ln(1 - p))/ t}$**, where** $\boldsymbol{t = 1}$

Section 3. Cost assumptions

A. ART

We obtained the cost of various potential antiretroviral agents used in ART from the Global Fund Pooled Procurement Price List, July 2020 [25]. For NNRTI-based ART, we considered two potential combinations of antiretroviral agents shown in Table S9 and S10, along with the monthly price of each of the relevant products: 1) abacavir (ABC)-lamivudine (3TC)-efavirenz (EFV); 2) zidovudine (AZT)-3TC-EFV.

We calculated an estimate of the average annual cost of NNRTI-based ART using the following approach:

1. First, for each of these two possible NNRTI-based regimens, we calculated the monthly cost of ART for four different weight ranges, based on the appropriate dosing for each weight range.
2. Next, for each of these two possible NNRTI-based regimens, we took the average of the monthly cost of ART of the four different ranges to obtain an estimate of the monthly cost of each of these two possible NNRTI-based regimens.
3. Third, we took the average of the monthly cost of each of these two possible NNRTI-based regimens to obtain an estimate of the monthly cost of NNRTI-based ART as a whole.
4. Finally, we converted this monthly cost to an annual cost, and we obtained an estimate of US$123 per person per year.

Table S9. Deriving cost of ABC-3TC-EFV ART

Weight (kg)	ABC + 3TC		EFV	
	Product	Tabs per day	Product	Tabs per day
10 – 13.9	Abacavir/Lamivudine 120/60mg tablet dispersible (30 tablets - $3.49)	2	Efavirenz 200mg tablet 30 ($1.50)	1
14 – 19.9		2.5		1.5
20 – 24.9		3		1.5
25 – 34.9	Abacavir/Lamivudine 600/300mg tablet 30 ($9.20)	1		2

Table S10. Deriving cost of AZT-3TC-EFV ART

Weight (kg)	AZT + 3TC		EFV	
	Product	Tabs per day	Product	Tabs per day
10 – 13.9	Lamivudine/Zidovudine 150/300mg tablet 60 ($5.25)	1	Efavirenz 200mg tablet 30 ($1.50)	1
14 – 19.9		1		1.5
20 – 24.9		1.5		1.5
25 – 34.9		2		2

For PI-based ART, we considered two potential combinations of antiretroviral agents shown in Table S11 and S12, along with the monthly price of each of the relevant products: 1) ABC-3TC-lopinavir/ritonavir (LPV/r); 2) AZT-3TC- LPV/r. We calculated an estimate of the average annual cost of PI-based ART using the same approach described above for NNRTI-based ART to arrive at an estimate of US$290 per person per year.

Table S11. Deriving cost of ABC-3TC-LPV/r

Weight (kg)	ABC + 3TC		LPV/r	
	Product	Tabs per day	Product	Packs per month
10 – 13.9	Abacavir/Lamivudine 120/60mg tablet dispersible 30 ($3.49)	2	Lopinavir/Ritonavir 80/20mg/ml oral solution 60ml*5 ($30.82)	2
14 – 19.9		2.5		2.5
20 – 24.9		3		3
25 – 34.9	Abacavir/Lamivudine 600/300mg tablet 30 ($9.20)	1		3

Table S12. Deriving cost of AZT-3TC-LPV/r

Weight (kg)	AZT + 3TC		LPV/r	
	Product	Tabs per day	Available Product	Packs per month
10 – 13.9	Lamivudine/Zidovudine 150/300mg tablet 60 ($5.25)	1	Lopinavir/Ritonavir 80/20mg/ml oral solution 60ml*5 ($30.82)	2
14 – 19.9		1		2.5
20 – 24.9		1.5		3
25 – 34.9		2		3

B. Inpatient days

In our model, the probability of a child living with HIV having a clinical event, including an opportunistic infection, depends on the child's CD4%/CD4 cell count. When a child does have a clinical event, we assume the patient is admitted to an inpatient health facility. To determine the number of inpatient days needed per clinical event, we extrapolate from South African data among adults living with HIV, which stratifies the number of inpatient days per hospitalization by CD4 cell count and ART status (**Table S13**) [26]. To calculate the total cost of care for a clinical event, the number of inpatient days required is multiplied by the unit cost of an inpatient day, which we assumed was $96.

Table S13. Average number of inpatient days per hospitalization by ART status and CD4 cell count. Adaption of Table 2 from Meyer-Rath et al [26].

CD4 Cell Count	Mean Length of Stay (days)
Pre-ART	
≤ 100 cells/mm³	9.8
101-200 cells/mm³	8.1
201-350 cells/mm³	7.8
>350 cells/mm³	6.4
On ART	
≤ 100 cells/mm³	12.3
101-200 cells/mm³	13.4
201-350 cells/mm³	9.5
>350 cells/mm³	7.0

While our analysis is intended to be broadly applicable to sub-Saharan Africa, no single base-case estimate will accurately capture the cost per inpatient day for all countries. We obtained estimates of the unit cost per inpatient day from five different countries representing a wide range of gross domestic product (GDP) per capita (Ghana, Kenya, Zambia, Uganda, and South Africa), shown in **Table S14** [27-31]. We converted estimates reported in the literature to US$2020 and used the average of these values as our base-case estimate (rounded to nearest dollar).

Table S14. Derivation of unit cost per inpatient day. Note: For South Africa, the original source reported estimates for care at different hospital levels: (primary ($60.89), secondary ($79.44), and tertiary ($108.51). The cost estimate reported for South Africa is based on the average of these values.

Country	Year Estimate Reported	Cost Estimate Reported (US$)	Consumer price index from reported year M01	Consumer price index from 2020 M01	Cost in 2020 US$ (adjusting for inflation	GDP per capita of country in 2019 in US$ (for reference)
South Africa[31]	2005	$82.95	51.78	113.8	$182.30	$6,001
Ghana[28]	2011	$39	42.26	113.9	$105.11	$2,202
Kenya[27]	2011	$41	110.57	201.57	$74.74	$1,817
Zambia[30]	2010	$20	105	246.72	$46.99	$1,305
Uganda[29]	2011	$41	105.23	180.26	$70.23	$794
				Average	$95.88	

We conducted a one-way sensitivity analysis of the unit cost per inpatient day ranging from US$15 to US$400 (base-case unit = US$96). This range was meant to capture uncertainty in both unit cost per inpatient day and the number of inpatient days per hospitalization, given that we extrapolated from adult data rather than directly using pediatric data, which is limited [26].

For example, the $15-unit cost scenario was meant to represent a situation in which the unit cost was only $30 (50% of base-case unit cost) and each hospitalization resulted in only 50% the number of inpatient days relative to those found in the adult South African study. Similarly, the $400-unit cost scenario was meant to represent a scenario in which the unit cost was $200 (double the base-base unit cost) and each hospitalization resulted in double the number of inpatient days relative to those found in the adult South African study. This approach is based on the mathematical relationship that doubling, or halving, the number of inpatient days per hospitalization has the same effect on total inpatient costs as doubling, or halving, the cost per inpatient day, respectively.

C. Outpatient visits

We assumed each child had an outpatient visit every 6 months. While our analysis is intended to be broadly applicable to sub-Saharan Africa, no single base-case estimate will accurately capture the cost per outpatient visit for all countries. We obtained estimates of the unit cost per outpatient visit from five different countries representing a wide range of gross domestic product (GDP) per capita (Ghana, Kenya, Zambia, Uganda, and South Africa), shown in **Table S15** [27-31]. We converted estimates reported in the literature to US$2020 and used the average of these values as our base-case estimate (rounded to nearest dollar).

Table S15. Derivation of unit cost per outpatient day.

Country	Year Estimate Reported	Cost Estimate Reported (US$)	Consumer price index from reported year M01	Consumer price index from 2020 M01	Cost in 2020 US$ (adjusting for inflation	GDP per capita of country in 2020 in US$ (for reference)
South Africa[31]	2005	$31.46	51.78	113.8	$69.14	$6,001
Ghana[28]	2011	$14	42.26	113.9	$37.73	$2,202
Kenya[27]	2011	$10	110.57	201.57	$18.23	$1,817
Zambia[30]	2010	$8	105	246.72	$18.80	$1,305
Uganda[29]	2011	$8	105.23	180.26	$13.70	$794
				Average	$31.52	

Note: For South Africa, the original source reported estimates for care at different outpatient levels: (primary ($20.90), secondary ($29.64), and tertiary ($43.84). The cost estimate reported for South Africa is based on the average of these values.

D. CD4 testing

To estimate the cost of CD4 cell count testing, we assume that CD4 cell count is checked at baseline and when virologic failure is detected to assess for risk of opportunistic infections [32]. Our unit cost estimate per CD4 cell count test of US$12 was obtained based on the price charged at the Coptic Hope Center in Nairobi, Kenya in 2019 [1]. This estimate is consistent with values used by other analyses, and it is also more recent than other references we found. For example, an analysis published by Phillips et al. in 2017 used a $10 unit cost per CD4 cell count test [33]. For this estimate, they cited two prior analyses: 1) Hyle et al., which was published in 2014, uses a $10 unit cost for a lab-based CD4 cell count test, and they cite a conference abstract from 2014, and a manuscript published in 2012 [34]; 2) Keebler et al., which was published in 2014, uses unit costs ranging from $8.28 to $9.50, based on estimates from 2011 and 2012 (found in Appendix) [35].

E. Viral load testing

Our base-case assumption about the cost per viral load test of US$54 is based on an estimate obtained from the Kenya Medical Research Institute (KEMRI) in 2019 [1]. In a one-way sensitivity analysis, we explore costs ranging from US$10 to US$80 per test. The lower end of this range was chosen to reflect announcements made by the Clinton Health Access Initiative regarding negotiations to make viral load testing available at a cost of US$12 per patient sample in several LMIC [36].

F. Resistance testing

Our base-case assumption about the cost per drug resistance test of US$125 is based on an estimate obtained from the Kenya Medical Research Institute (KEMRI) in 2019 [1]. For comparison, Phillips et al. assumes a drug resistance test in sub-Saharan Africa costs US$100, based on "Consensus from World Health Organisation HIV Resistance Network (HIVResNet)" [33].

Section 4. Model calibration

A. Overview

Model calibration is the process of determining which combination of possible parameter values, within empirically-derived confidence intervals, are most consistent with observed outcome data [37]. Our model calibration led to the parameter values described in Section 1 of this Appendix, which are the parameter values we used in our model-based cost-effectiveness analysis. In this section (Section 4), we describe our model calibration process (**Figure S1**).

Our model calibration process included three main groups of data targets:

1. Survival from birth to 60 months among perinatally-infected children without ART
2. Rates of mortality and clinical events among children on ART
3. CD4% to CD4 cell count conversion targets

Table S16 includes all model parameters whose values were informed by our model calibration and describes the ranges of values and probability distributions we assigned to each parameter. We generated 20,000 parameter sets, each with a unique combination of values for the parameters in **Table S16**. Each time a parameter set was generated, for each parameter, a value was drawn at random from the probability distributions we assigned to it. This process was repeated 20,000 times to generate the 20,000 unique parameter sets.

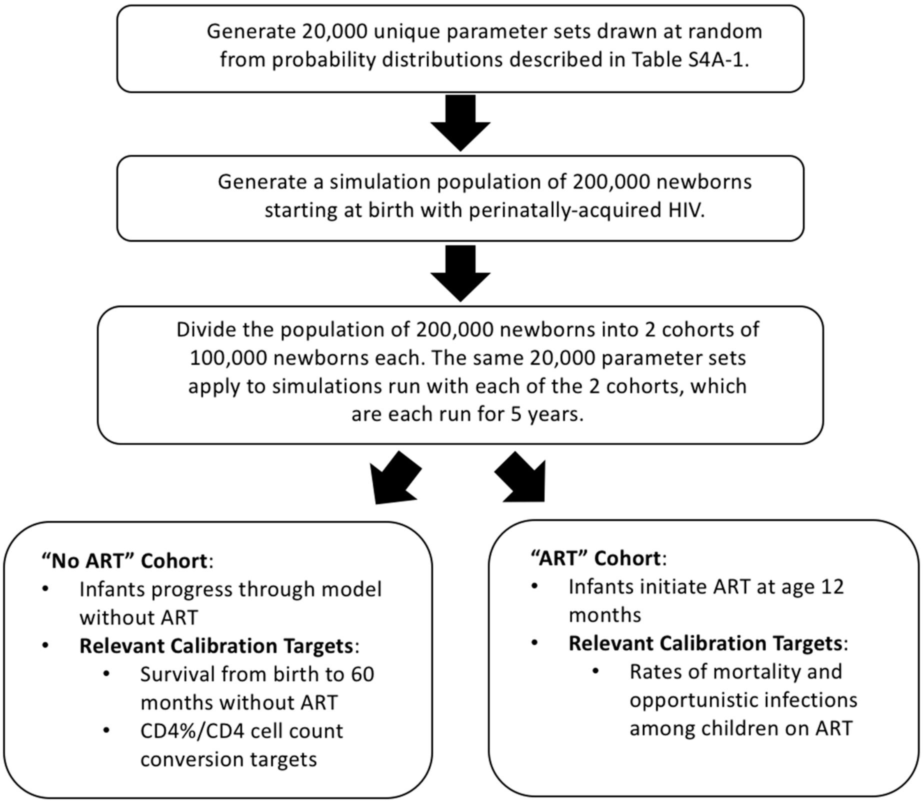

Figure S1. Overview of Model Calibration.

Table S16. Probability distributions for parameters adjusted in model calibration.

**Input parameters for model calibration**	**Sampling Distribution**	**Distribution parameters (range for uniform; mean, SD for normal distribution)**
**Initial CD4% distribution at birth**		
Initial mean of CD4% at age 0	Uniform	42-50%
Initial SD of CD4% at age 0	Uniform	9.40-14.10%
**CD4% monthly decline**		
Age ≤ 3 months	Uniform	3.00-8.00%
Age 4 months - 5 years	Uniform	0.30-0.70%
**Mortality**		
Monthly probability of death with no history of clinical event [a]		
CD4% < 15	Normal	0.40%, 0.02%
CD4% 15-24	Normal	0.40%, 0.02%
CD4% ≥ 25	Normal	0.40%, 0.02%
Probability of death within 30 days of clinical events [a]	Normal	3.10%, 0.05%
Monthly probability of death with history of clinical event (> 30 days post-event) [a]		
CD4% < 15	Normal	2.40%, 0.05%
CD4% 15-24	Normal	0.80%, 0.03%
CD4% ≥ 25	Normal	0.40%, 0.02%

**Immunologic recovery on ART**		
Relative risk reduction in event risk for children on ART		
Clinical event	Uniform	0.85-1
Mortality	Uniform	0.90-1
CD4% monthly increase in children < 5 years of age		
NNRTI in the first 6 months	Normal	2.20%, 0.17%
NNRTI after 6 months	Normal	0.70%, 0.05%
PI in the first 6 months	Normal	1.90%, 0.09%
PI after 6 months	Normal	0.40%, 0.02%
**CD4 cell count distribution at 5 years (used for CD4% to absolute conversion)**		
Mean of CD4 cell count at age 5 years	Uniform	301-559
SD of CD4 cell count at age 5 years	Uniform	280-520

[a] The three different categories of parameters above related to monthly probability of death are likely underestimates, as explained in detail in Section 4Ci. Thus, they were multiplied by age-specific multipliers presented in Table S18, which were adjusted in model calibration but presented in a table separate from this one due to space limitations. The data sources we used to inform these probability distributions are described in Section 4B. SD = standard deviation;

Calibration with mean square error approach

Each of the two cohorts were run simultaneously over a 5-year time horizon to calibrate against all three groups of calibration data targets simultaneously, using the following approach:

1. For each of the calibration targets, we set minimum and maximum bounds for each of the, which are described in detail below in their respective sub-sections in Section 4C.
2. For each of the calibration targets, we calculated the error between the target and its corresponding model outcome. The error was normalized by taking the square of the error as follows: Square error = ((model outcome – target) / (maximum bound – minimum bound))^2
3. For each of the 20,000 parameter sets, we took the mean of the square error for each of the calibration targets to calculate the mean square error (MSE). The MSE indicates how well model output match the three groups of calibration targets as a whole (parameter sets with lower MSEs indicate a better fit compared to parameter sets with higher MSEs).
4. After running the calibration simulations with all 20,000 parameter sets, we removed parameter sets with outcomes that were outside the minimum/maximum bounds of any of the calibration targets. We ranked the remaining parameter sets in order of lowest to highest MSE. We chose the parameter set with the lowest MSE as the final parameter set we used for our model-based analyses.

Section 4B below describes the data sources to inform the probability distributions we assigned to the parameters in **Table S16**. Section 4C below provides a detailed description of each of the three groups of data targets. This includes an explanation of the assumptions we made for each relevant simulation cohort ("no ART" vs. "ART").

B. Data sources used to inform parameter values

Initial CD4% and rate of decline of CD4%

The range of values we explored in our model calibration for initial CD4% at birth (mean and standard deviation) were informed by an analysis conducted by Ciaranello et al. (results

presented in their Table 1) [6], using data from the Women and Infants Transmission Study (WITS). Although it is a U.S.-based study, it was chosen because it has appropriate longitudinal data for CD4%. Similar to Ciaranello et al. [6], we explored more rapid rates of CD4% decline during the first 3 months of life to reflect trends that have been observed in this age group.

Probability of death

The ranges we explored for our model parameters related to the probability of death were informed by Ciaranello et al. (results presented in their Table 1) [6].

Parameters related to immunologic recovery on ART

The values we explored in our model calibration for the relative risk reduction of clinical event and death with ART, as well as for CD4% monthly increase on effective ART, were informed by values used by Ciaranello et al. [8].

CD4 conversion

We reviewed the literature to identify studies from sub-Saharan Africa with data on CD4% and/or CD4 cell count for children near the age of 5 years prior to initiating ART. The data we found is summarized in **Table S17.** Median CD4% and median CD4 cell count at age 5 years were two data targets in our model calibration. The values for these targets were informed by the data presented in **Table S17**.

Table S17. Pre-ART CD4% and CD4 cell count near age 5 years. Calibration targets at the bottom of this table were informed by the data presented here.

**Age (years) Median (25$^{\%tile}$-75$^{\%tile}$)**	**CD4% Median (25$^{\%tile}$-75$^{\%tile}$)**	**CD4 cell count (cells/µl) Median (25$^{\%tile}$-75$^{\%tile}$)**	**Study Location**	**Source**
7.1 (3.6, 4.2)	11.9 (5.8, 17.5)	356 (132, 603)	Zambia	Sutcliffe et al.[38]
6.4 (3.5, 9.6)	12 (7, 17)		South Africa	Fatti et al.[39]
4.83 (1.7, 9.08)	14 (8.9, 20)	381 (180, 734)	South Africa	Davies et al.[40]
7.6 (4.5, 11)	17 (7.5, 28)	397 (183, 800)	South Africa	Muenchhoff et al.[41]
6.3 (2.4, 9.7)	12 (6, 17)		Uganda, Zimbabwe	Prendergast et al. [42]
**Calibration targets**	12 (6, 17)	370 (160, 700)		

In order to convert CD4% at 5 years of age to CD4 cell count for each child, a "CD4 cell count distribution" was needed, as described in Section 1E. The "CD4 cell count distribution" described in Section 1E was the distribution from the final parameter set chosen in the model calibration (from the 20,000 possible parameter sets). Thus, this parameter was included and adjusted in the model calibration process, and the range of values we explored is described in **Table S16**. We treated mean and standard deviation (SD) as two separate parameters. For the mean, we explored a uniform distribution ranging from 301-559, and for the SD, we explored a uniform distribution ranging from 280-520.

To select these uniform distributions, described in **Table S16,** for the mean and SD of the "CD4 cell count distribution at 5 years" parameter, we used the following approach:

1. First, we assumed CD4 cell count at age 5 years follows a normal distribution.
2. Next, we aimed to solve for the mean and SD of this normal distribution by using the two following algebraic equations.

   Mean – 0.675 x SD = 160 (equation for 25th percentile of normal distribution)

   Mean + 0.675 x SD = 700 (equation for 75th percentile of normal distribution)

   The solutions to these two equations are: **Mean = 430** and **SD = 400**.
3. Finally, we obtained the ranges to explore in a uniform distribution for the mean of CD4 cell count distribution using the mean of 430 +/- 30%, which yields the range of 301- 559. We obtained

the ranges to explore in a uniform distribution for SD of the CD4 cell count distribution by using the SD of 400 +/- 30%, which yields the range of 280 – 520.

C. Calibration targets

i. Survival from birth to 60 months without ART

Description of calibration target data source

Our calibration targets included survival curves published by UNAIDS that included >1,300 children from eight sub-Saharan African countries with untreated, perinatally-acquired HIV. These UNAIDS survival curves were also used in the development and calibration of the CEPAC-Pediatric model [6]. We attempted to obtain the UNAIDS data directly from all the sources cited and used by Ciaranello et al. [6]. However, we were only able to find three of the four that were cited [43-45]. Marston et al. provided mortality data at 6 months, 12 months, and 24 months of age only, and it matched the respective data presented by Ciaranello et al. [6,43]. The fourth citation was a UNAIDS Child Survival Working Group policy report published in 2010 that we could no longer find on the internet, and we assume that this report provided the remaining mortality data for 36 months, 48 month, and 60 months of age. Therefore, we relied upon the UNAIDS survival curves for 36-60 months of age as presented by Ciaranello et al. [6].

Approach for calibration simulation

Survival curves from ages 0 to 60 months from the UNAIDS cohort was ideal as a calibration target because this data was collected before ART was widely available for children. In contrast, children from the East Africa IeDEA cohort likely received ART when they needed it. Although the Ciaranello 2014 analysis censored data once ART initiation occurred, estimates of the probability of clinical events and death (those presented in our Table S16) are likely underestimates due to two reasons. First, because subsequent data was censored once a child initiated ART, this eliminated the ability to observe the risks of clinical events and death that might otherwise occur in the absence of ART. Second, the IeDEA cohort may be over-representative of children who are long-term non-progressors [46], and thus have some level of survivor bias.

In order to adjust for this underestimation, we used the following approach: In the model calibration, each time a parameter set (of the 20,000) was generated, a value was drawn at random from the mortality probability distributions described in Table S16, and a value was also drawn at random from the distributions for their corresponding multipliers in Table S18. For each parameter set, we multiplied the monthly probabilities of death (after converting them to monthly rates) in Table S16 by their corresponding multipliers in Table S18. The mortality parameter values in the final parameter set used in the model that are described in Table S1 are the product of these two factors. The CEPAC-Pediatrics model used a similar mortality multiplier approach [6]. Our survival calibration simulation also accounted for the non-HIV background mortality rate in the UNAIDS cohort [6].

Table S18. Uniform distributions used for mortality multipliers in model calibration.

	**Sampling Distribution**	**Ranges for varying**
Multipliers for monthly probability of death with no history of clinical event		
Age 0-6 months	Uniform	1-20
Age 7-12 months	Uniform	1-20
Age 13-24 months	Uniform	0.5-5
Age 25-36 months	Uniform	0.5-5
Age 37-48 months	Uniform	0.2-2
Age 49-60 months	Uniform	0.2-2
Multipliers for probability of death within 30 days of clinical events	Uniform	0.5-5

Multipliers for monthly probability of death with history of clinical event (> 30 days post-event)		
Age 0-6 months	Uniform	1-20
Age 7-12 months	Uniform	1-20
Age 13-24 months	Uniform	0.5-5
Age 25-36 months	Uniform	0.5-5
Age 37-48 months	Uniform	0.2-2
Age 49-60 months	Uniform	0.2-2

Comparing model output to calibration targets

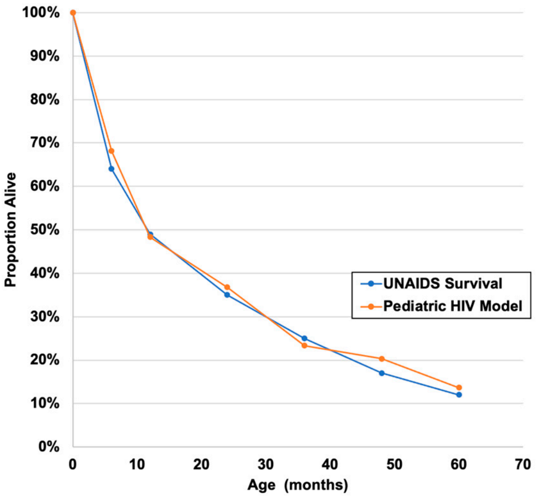

Figure S2. Survival (0 – 60 months) model output and calibration targets. The minimum and maximum calibration bounds for each calibration target data point was +/- 10% (at 6 months, 12 months, 24 months, 36 months, 48 months, and 60 months).

ii. Mortality and clinical event rates on ART

Description of calibration target data source

The data for the mortality rate and opportunistic infection (OI) rate calibration targets we used are from the International Maternal, Pediatric, and Adolescent Clinical Trial (IMPAACT) P1060 study [47,48]. We obtained these rates directly from Ciaranello et al.'s study, which derived these rates with access to primary data from the P1060 trial [8].

Approach for calibration simulation

We compared mortality and OI rates among children from our "ART" cohort to the mortality and OI rate calibration targets. At the start of the simulation with the 100,000 newborns in the "ART" cohort, 50,000 newborns were randomized into the nevirapine (NVP) cohort, and 50,000 newborns were randomized into the lopinavir (LPV) cohort. In the NVP cohort, infants received NVP-based ART as the first-line regimen and LPV-based ART as the second-line regimen. In the LPV cohort, infants received LPV-based ART as the first-line regimen and NVP-based ART as the second-line regimen. These two sub-cohorts (NVP and LPV cohorts) were meant to simulate the randomization to LPV-based ART vs. NVP-based ART that occurred in the P1060 trial. In the simulation, children do not initiate their first-line ART regimen until they reach 12 months of age. We were unable to obtain rates of virologic failure directly from the P1060 manuscript, as virologic failure and mortality were presented as a combined single outcome [47,48]. **Table S19** below presents the proportion of patients with virologic suppression at 48 weeks on their respective ART regimens, as presented by Ciaranello et al. in Appendix Table A,

which they calculated with access to the primary data. We subtract the proportion with viral suppression for each regimen in **Table S19** from 1 to derive the probability of virologic failure over 12 months. We assumed a child could have virologic failure during the first 12 months on an ART regimen. We assume viral load testing occurs at 6 months after ART initiation and every 12 months thereafter, and we assume that regimen switching occurs immediately for 100% of children diagnosed with virologic failure.

Table S19. Proportion of children with viral suppression after 48 weeks.

	**NVP Cohort**	**LPV Cohort**
First-line ART	72%<br>(nevirapine)	86%<br>(lopinavir)
Second-line ART	70%<br>(lopinavir)	71%<br>(nevirapine)

In our calibration simulation, the probability is virologic failure over 12 months for each respective regimen = 1 – proportion with viral suppression.

Comparing model output to calibration targets

Mortality rate from the P1060 trial, as derived by Ciaranello et al. (their Appendix Table B and Appendix Table C), was as 3.29 per 100 person-years (PY), observed over a median follow-up time of 72 weeks. The rates of WHO3, WHO4, and TB clinical events were 9.30/100PY, 0.73/100PY, and 5.60/100PY, respectively (did not specify the follow-up time) [8]. Because our model simulates the occurrence of clinical events as a whole, rather than WHO 3, WHO 4, and TB separately, we used the average of these three rates as our target for rate of clinical events (5.21/100PY). We calculated the mortality rate (3.34/100PY) and clinical event rate (5.15/100PY) in our simulation over the first two years after ART was initiated in our "ART" cohort at 12 months of age and compared these values to their respective targets (**Table S20).**

Table S20. Mortality and clinical event rate model output and calibration targets.

	**Final model parameter set**	**P1060 target**	**Minimum target bound**	**Maximum target bound**
Mortality rate<br>(per 100 person-years)	3.34	3.29	2.0	5.0
Clinical event rate<br>(per 100 person-years)	5.15	5.21	0.73	9.3

iii. CD4%/CD4 cell count conversion

Table S21. CD4% and CD4 cell count at age 5 years model output and calibration targets. Square errors were calculated for all 6 outcomes shown here (CD4% median, CD4% $25^{\%tile}$, CD4% $75^{\%tile}$, CD4 cell count median, CD4 cell count $25^{\%tile}$, and CD4 cell count $75^{\%tile}$.

	**CD4% median**	**CD4% $25^{\%tile}$**	**CD4% $75^{\%tile}$**	**CD4 cell count median**	**CD4 cell count $25^{\%tile}$**	**CD4 cell count $75^{\%tile}$**
Final values	9.23	2.33	15.34	496.13	281.71	729.62
Target	12	6	17	370	160	700
Minimum Bound		1			100	
Maximum Bound		40			1200	

Section 5. Additional results not included in manuscript

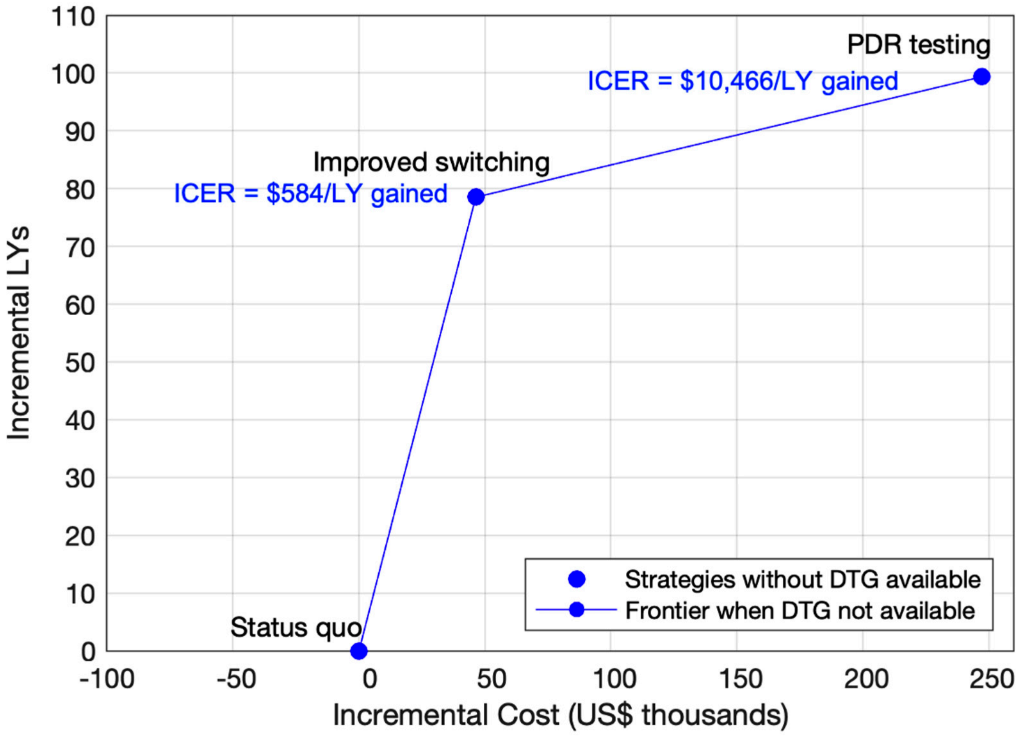

Figure S3. **Incremental costs and health benefits of strategies compared to the *status quo*, when we assume the probability of switching to second-line ART when virologic failure is diagnosed improves to 60% (instead of 80%) with the *improved switching* strategy.** Incremental costs and health benefits are per 1,000 children initiating ART over a 10-year time horizon.

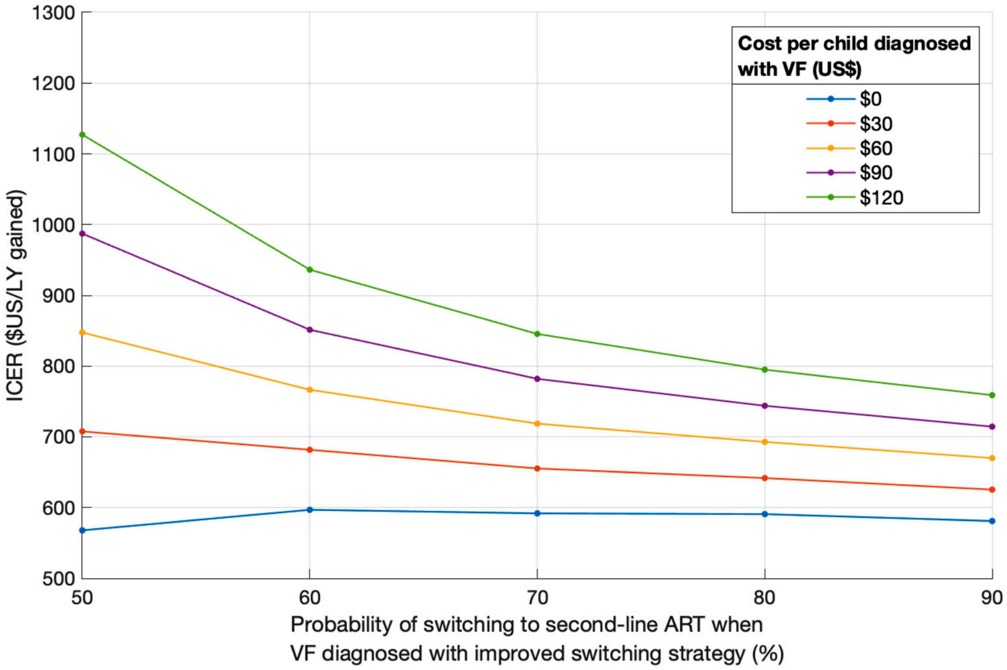

Figure S4. **Cost-effectiveness of *DTG improved switching* strategy relative to *DTG status quo* over a range of strategy effectiveness and cost per child diagnosed with VF.**
VF = virologic failure

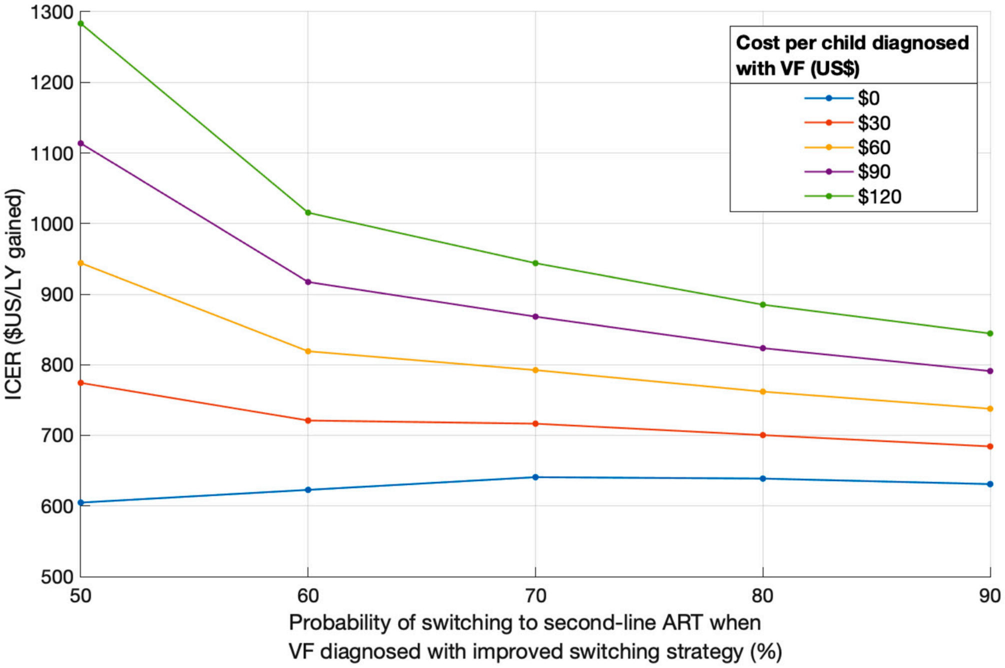

Figure S5. **Cost-effectiveness of *DTG improved switching* strategy relative to *DTG status quo* over a range of strategy effectiveness and cost per child diagnosed with VF, in a scenario in which the probability of virologic failure with PI-based second line ART is 40% over 24 months.** VF = virologic failure

References

1. Duarte, H.A.; Babigumira, J.B.; Enns, E.A.; Stauffer, D.C.; Shafer, R.W.; Beck, I.A.; Garrison, L.P., Jr.; Chung, M.H.; Frenkel, L.M.; Bendavid, E. Cost-effectiveness analysis of pre-ART HIV drug resistance testing in Kenyan women. *EClinicalMedicine* **2020**, *22*, 100355, doi:10.1016/j.eclinm.2020.100355.
2. Department of Health and Human Services. *Guidelines for the Prevention and Treatment of Opportunistic Infections in*
HIV-Exposed and HIV-Infected Children. Panel on Opportunistic Infections in HIV-Exposed and HIV-Infected Children.; 2013.
3. Ciaranello, A.; Lu, Z.; Ayaya, S.; Losina, E.; Musick, B.; Vreeman, R.; Freedberg, K.A.; Abrams, E.J.; Dillabaugh, L.; Doherty, K., et al. Incidence of World Health Organization stage 3 and 4 events, tuberculosis and mortality in untreated, HIV-infected children enrolling in care before 1 year of age: an IeDEA (International Epidemiologic Databases To Evaluate AIDS) East Africa regional analysis. *Pediatr Infect Dis J* **2014**, *33*, 623-629, doi:10.1097/INF.0000000000000223.
4. Desmonde, S.; Neilan, A.M.; Musick, B.; Patten, G.; Chokephaibulkit, K.; Edmonds, A.; Duda, S.N.; Malateste, K.; Wools-Kaloustian, K.; Ciaranello, A.L., et al. Time-varying age- and CD4-stratified rates of mortality and WHO stage 3 and stage 4 events in children, adolescents and youth 0 to 24 years living with perinatally acquired HIV, before and after

antiretroviral therapy initiation in the paediatric IeDEA Global Cohort Consortium. *J Int AIDS Soc* **2020**, *23*, e25617, doi:10.1002/jia2.25617.
5. United Nations, D.o.E.a.S.A., Population Division (2019). World Population Prospects 2019, Online Edition. Rev. Availabe online: https://population.un.org/wpp/Download/Standard/Population/ (accessed on 15 November 2020)).
6. Ciaranello, A.L.; Morris, B.L.; Walensky, R.P.; Weinstein, M.C.; Ayaya, S.; Doherty, K.; Leroy, V.; Hou, T.; Desmonde, S.; Lu, Z., et al. Validation and calibration of a computer simulation model of pediatric HIV infection. *PLoS One* **2013**, *8*, e83389, doi:10.1371/journal.pone.0083389.
7. Institute for Health Metrics and Evaluation. GBD Compare Data Visualization, October 15, 2020. Availabe online: https://vizhub.healthdata.org/gbd-compare/ (accessed on 15 November, 2020)).
8. Ciaranello, A.L.; Doherty, K.; Penazzato, M.; Lindsey, J.C.; Harrison, L.; Kelly, K.; Walensky, R.P.; Essajee, S.; Losina, E.; Muhe, L., et al. Cost-effectiveness of first-line antiretroviral therapy for HIV-infected African children less than 3 years of age. *AIDS* **2015**, *29*, 1247-1259, doi:10.1097/QAD.0000000000000672.
9. Collaborative Initiative for Paediatric, H.I.V.E.; Research Global Cohort, C. Incidence of switching to second-line antiretroviral therapy and associated factors in children with HIV: an international cohort collaboration. *Lancet HIV* **2019**, *6*, e105-e115, doi:10.1016/S2352-3018(18)30319-9.
10. Boerma, R.S.; Boender, T.S.; Bussink, A.P.; Calis, J.C.; Bertagnolio, S.; Rinke de Wit, T.F.; Boele van Hensbroek, M.; Sigaloff, K.C. Suboptimal Viral Suppression Rates Among HIV-Infected Children in Low- and Middle-Income Countries: A Meta-analysis. *Clin Infect Dis* **2016**, *63*, 1645-1654, doi:10.1093/cid/ciw645.
11. Madec, Y.; Leroy, S.; Rey-Cuille, M.A.; Huber, F.; Calmy, A. Persistent difficulties in switching to second-line ART in sub-saharan Africa--a systematic review and meta-analysis. *PLoS One* **2013**, *8*, e82724, doi:10.1371/journal.pone.0082724.
12. Haas, A.D.; Keiser, O.; Balestre, E.; Brown, S.; Bissagnene, E.; Chimbetete, C.; Dabis, F.; Davies, M.A.; Hoffmann, C.J.; Oyaro, P., et al. Monitoring and switching of first-line antiretroviral therapy in adult treatment cohorts in sub-Saharan Africa: collaborative analysis. *Lancet HIV* **2015**, *2*, e271-278, doi:10.1016/S2352-3018(15)00087-9.
13. Davies, M.A.; Moultrie, H.; Eley, B.; Rabie, H.; Van Cutsem, G.; Giddy, J.; Wood, R.; Technau, K.; Keiser, O.; Egger, M., et al. Virologic failure and second-line antiretroviral therapy in children in South Africa--the IeDEA Southern Africa collaboration. *J Acquir Immune Defic Syndr* **2011**, *56*, 270-278, doi:10.1097/QAI.0b013e3182060610.
14. Wools-Kaloustian, K.; Marete, I.; Ayaya, S.; Sohn, A.H.; Van Nguyen, L.; Li, S.; Leroy, V.; Musick, B.S.; Newman, J.E.; Edmonds, A., et al. Time to First-Line ART Failure and Time to Second-Line ART Switch in the IeDEA Pediatric Cohort. *J Acquir Immune Defic Syndr* **2018**, *78*, 221-230, doi:10.1097/QAI.0000000000001667.
15. Boerma, R.S.; Sigaloff, K.C.; Akanmu, A.S.; Inzaule, S.; Boele van Hensbroek, M.; Rinke de Wit, T.F.; Calis, J.C. Alarming increase in pretreatment HIV drug resistance in children

living in sub-Saharan Africa: a systematic review and meta-analysis. *J Antimicrob Chemother* **2017**, *72*, 365-371, doi:10.1093/jac/dkw463.
16. WHO. *HIV Drug Resistance Report 2019*; Geneva, Switzerland., 2019.
17. Jain, V.; Sucupira, M.C.; Bacchetti, P.; Hartogensis, W.; Diaz, R.S.; Kallas, E.G.; Janini, L.M.; Liegler, T.; Pilcher, C.D.; Grant, R.M., et al. Differential persistence of transmitted HIV-1 drug resistance mutation classes. *J Infect Dis* **2011**, *203*, 1174-1181, doi:10.1093/infdis/jiq167.
18. Kityo, C.; Boerma, R.S.; Sigaloff, K.C.E.; Kaudha, E.; Calis, J.C.J.; Musiime, V.; Balinda, S.; Nakanjako, R.; Boender, T.S.; Mugyenyi, P.N., et al. Pretreatment HIV drug resistance results in virological failure and accumulation of additional resistance mutations in Ugandan children. *J Antimicrob Chemother* **2017**, *72*, 2587-2595, doi:10.1093/jac/dkx188.
19. Hamers, R.L.; Schuurman, R.; Sigaloff, K.C.; Wallis, C.L.; Kityo, C.; Siwale, M.; Mandaliya, K.; Ive, P.; Botes, M.E.; Wellington, M., et al. Effect of pretreatment HIV-1 drug resistance on immunological, virological, and drug-resistance outcomes of first-line antiretroviral treatment in sub-Saharan Africa: a multicentre cohort study. *Lancet Infect Dis* **2012**, *12*, 307-317, doi:10.1016/S1473-3099(11)70255-9.
20. Chung, M.H.; McGrath, C.J.; Beck, I.A.; Levine, M.; Milne, R.S.; So, I.; Andersen, N.; Dross, S.; Coombs, R.W.; Chohan, B., et al. Evaluation of the management of pretreatment HIV drug resistance by oligonucleotide ligation assay: a randomised controlled trial. *Lancet HIV* **2019**, 10.1016/S2352-3018(19)30337-6, doi:10.1016/S2352-3018(19)30337-6.
21. Dugdale, C.M.; Ciaranello, A.L.; Bekker, L.G.; Stern, M.E.; Myer, L.; Wood, R.; Sax, P.E.; Abrams, E.J.; Freedberg, K.A.; Walensky, R.P. Risks and Benefits of Dolutegravir- and Efavirenz-Based Strategies for South African Women With HIV of Child-Bearing Potential: A Modeling Study. *Ann Intern Med* **2019**, 10.7326/M18-3358, doi:10.7326/M18-3358.
22. Boerma, R.S.; Bunupuradah, T.; Dow, D.; Fokam, J.; Kariminia, A.; Lehman, D.; Kityo, C.; Musiime, V.; Palumbo, P.; Schoffelen, A., et al. Multicentre analysis of second-line antiretroviral treatment in HIV-infected children: adolescents at high risk of failure. *J Int AIDS Soc* **2017**, *20*, 21930, doi:10.7448/IAS.20.1.21930.
23. WHO. *Update of recommendations on first- and second-line antiretroviral regimens. July 2019.*; Geneva: World Health Organization, 2019.
24. Carlucci, J.G.; Liu, Y.; Clouse, K.; Vermund, S.H. Attrition of HIV-positive children from HIV services in low and middle-income countries. *AIDS* **2019**, *33*, 2375-2386, doi:10.1097/QAD.0000000000002366.
25. Global Fund. Global Fund Pooled Procurement Price List July 2020. Availabe online: https://www.theglobalfund.org/media/5813/ppm_arvreferencepricing_table_en.pdf (accessed on 1 August 2020).
26. Meyer-Rath, G.; Brennan, A.T.; Fox, M.P.; Modisenyane, T.; Tshabangu, N.; Mohapi, L.; Rosen, S.; Martinson, N. Rates and cost of hospitalization before and after initiation of antiretroviral therapy in urban and rural settings in South Africa. *J Acquir Immune Defic Syndr* **2013**, *62*, 322-328, doi:10.1097/QAI.0b013e31827e8785.
27. Institute for Health Metrics and Evaluation (IHME). *Health Service Provision in Kenya:Assessing Facility Capacity, Costs of Care, and Patient Perspectives*; IHME: Seattle, WA, 2014.

28. Institute for Health Metrics and Evaluation (IHME). *Health Service Provision in Ghana:Assessing Facility Capacity, Costs of Care, and Patient Perspectives*; IHME: Seattle, WA, 2015.
29. Institute for Health Metrics and Evaluation (IHME). *Health Service Provision in Uganda:Assessing Facility Capacity, Costs of Care, and Patient Perspectives*; IHME: Seattle, WA, 2014.
30. Institute for Health Metrics and Evaluation (IHME). *Health Service Provision in Zambia:Assessing Facility Capacity, Costs of Care, and Patient Perspectives*; IHME: Seattle, WA, 2014.
31. WHO-CHOICE. Estimates of Unit Costs for Patient Services for South Africa. Availabe online: https://www.who.int/choice/country/zaf/cost/en/ (accessed on 23 November 2019)).
32. Kenya Ministry of Health. *Guidelines on Use of Antiretroviral Drugs for Treating and Preventing HIV Infection in Kenya. 2018 Edition*; NASCOP: Nairobi, Kenya, August 2018, 2018.
33. Phillips, A.N.; Cambiano, V.; Nakagawa, F.; Revill, P.; Jordan, M.R.; Hallett, T.B.; Doherty, M.; De Luca, A.; Lundgren, J.D.; Mhangara, M., et al. Cost-effectiveness of public-health policy options in the presence of pretreatment NNRTI drug resistance in sub-Saharan Africa: a modelling study. *Lancet HIV* **2018**, *5*, e146-e154, doi:10.1016/S2352-3018(17)30190-X.
34. Hyle, E.P.; Jani, I.V.; Lehe, J.; Su, A.E.; Wood, R.; Quevedo, J.; Losina, E.; Bassett, I.V.; Pei, P.P.; Paltiel, A.D., et al. The clinical and economic impact of point-of-care CD4 testing in mozambique and other resource-limited settings: a cost-effectiveness analysis. *PLoS Med* **2014**, *11*, e1001725, doi:10.1371/journal.pmed.1001725.
35. Keebler, D.; Revill, P.; Braithwaite, S.; Phillips, A.; Blaser, N.; Borquez, A.; Cambiano, V.; Ciaranello, A.; Estill, J.; Gray, R., et al. Cost-effectiveness of different strategies to monitor adults on antiretroviral treatment: a combined analysis of three mathematical models. *Lancet Glob Health* **2014**, *2*, e35-43, doi:10.1016/S2214-109X(13)70048-2.
36. Clinton Health Access Initiative. *HIV Market Report: The state of the HIV treatment, testing, and prevention markets in low- and middle-income countries, 2017-2022. Issue 9, September 2018.*; 2018.
37. Hunink, M.G.M., Weinstein, M.C., Wittenberg, E., Drummond, M.F., Pliskin, J.S., Wong, J.B., Glasziou, P.P. *Decision Making in Health and Medicine: Integrating Evidence and Values*, 2 ed.; Cambridge University Press: Cambridge, United Kingdom, 2014.
38. Sutcliffe, C.G.; Bolton-Moore, C.; van Dijk, J.H.; Cotham, M.; Tambatamba, B.; Moss, W.J. Secular trends in pediatric antiretroviral treatment programs in rural and urban Zambia: a retrospective cohort study. *BMC Pediatr* **2010**, *10*, 54, doi:10.1186/1471-2431-10-54.
39. Fatti, G.; Bock, P.; Eley, B.; Mothibi, E.; Grimwood, A. Temporal trends in baseline characteristics and treatment outcomes of children starting antiretroviral treatment: an analysis in four provinces in South Africa, 2004-2009. *J Acquir Immune Defic Syndr* **2011**, *58*, e60-67, doi:10.1097/QAI.0b013e3182303c7e.
40. Davies, M.A.; Phiri, S.; Wood, R.; Wellington, M.; Cox, V.; Bolton-Moore, C.; Timmerman, V.; Moultrie, H.; Ndirangu, J.; Rabie, H., et al. Temporal trends in the characteristics of children at antiretroviral therapy initiation in southern Africa: the IeDEA-SA Collaboration. *PLoS One* **2013**, *8*, e81037, doi:10.1371/journal.pone.0081037.

41. Muenchhoff, M.; Adland, E.; Roider, J.; Kloverpris, H.; Leslie, A.; Boehm, S.; Keppler, O.T.; Ndung'u, T.; Goulder, P.J.R. Differential Pathogen-Specific Immune Reconstitution in Antiretroviral Therapy-Treated Human Immunodeficiency Virus-Infected Children. *J Infect Dis* **2019**, *219*, 1407-1417, doi:10.1093/infdis/jiy668.
42. Prendergast, A.J.; Szubert, A.J.; Berejena, C.; Pimundu, G.; Pala, P.; Shonhai, A.; Musiime, V.; Bwakura-Dangarembizi, M.; Poulsom, H.; Hunter, P., et al. Baseline Inflammatory Biomarkers Identify Subgroups of HIV-Infected African Children With Differing Responses to Antiretroviral Therapy. *J Infect Dis* **2016**, *214*, 226-236, doi:10.1093/infdis/jiw148.
43. Marston, M.; Becquet, R.; Zaba, B.; Moulton, L.H.; Gray, G.; Coovadia, H.; Essex, M.; Ekouevi, D.K.; Jackson, D.; Coutsoudis, A., et al. Net survival of perinatally and postnatally HIV-infected children: a pooled analysis of individual data from sub-Saharan Africa. *Int J Epidemiol* **2011**, *40*, 385-396, doi:10.1093/ije/dyq255.
44. Becquet, R.; Marston, M.; Dabis, F.; Moulton, L.H.; Gray, G.; Coovadia, H.M.; Essex, M.; Ekouevi, D.K.; Jackson, D.; Coutsoudis, A., et al. Children who acquire HIV infection perinatally are at higher risk of early death than those acquiring infection through breastmilk: a meta-analysis. *PLoS One* **2012**, *7*, e28510, doi:10.1371/journal.pone.0028510.
45. Newell, M.L.; Coovadia, H.; Cortina-Borja, M.; Rollins, N.; Gaillard, P.; Dabis, F.; Ghent International, A.S.W.G.o.H.I.V.I.i.W.; Children. Mortality of infected and uninfected infants born to HIV-infected mothers in Africa: a pooled analysis. *Lancet* **2004**, *364*, 1236-1243, doi:10.1016/S0140-6736(04)17140-7.
46. Warszawski, J.; Lechenadec, J.; Faye, A.; Dollfus, C.; Firtion, G.; Meyer, L.; Douard, D.; Monpoux, F.; Tricoire, J.; Benmebarek, Y., et al. Long-term nonprogression of HIV infection in children: evaluation of the ANRS prospective French Pediatric Cohort. *Clin Infect Dis* **2007**, *45*, 785-794, doi:10.1086/521165.
47. Violari, A.; Lindsey, J.C.; Hughes, M.D.; Mujuru, H.A.; Barlow-Mosha, L.; Kamthunzi, P.; Chi, B.H.; Cotton, M.F.; Moultrie, H.; Khadse, S., et al. Nevirapine versus ritonavir-boosted lopinavir for HIV-infected children. *N Engl J Med* **2012**, *366*, 2380-2389, doi:10.1056/NEJMoa1113249.
48. Palumbo, P.; Lindsey, J.C.; Hughes, M.D.; Cotton, M.F.; Bobat, R.; Meyers, T.; Bwakura-Dangarembizi, M.; Chi, B.H.; Musoke, P.; Kamthunzi, P., et al. Antiretroviral treatment for children with peripartum nevirapine exposure. *N Engl J Med* **2010**, *363*, 1510-1520, doi:10.1056/NEJMoa1000931.
